# Supplementary material for: Dopamine-sensitive neurons in the mesencephalic locomotor region control locomotion initiation, stop, and turns
Source: Cell Rep. 2024 May 8;43(5):114187. doi: 10.1016/j.celrep.2024.114187 (PMC11157412; doi:10.1016/j.celrep.2024.114187)
Supplement: Document S1. Figures S1–S8 [file mmc1.pdf]

Cell Reports, Volume *43*

Supplemental information

Dopamine-sensitive neurons in the mesencephalic locomotor region control locomotion initiation, stop, and turns

Andrea Juárez Tello, Cornelis Immanuel van der Zouwen, Léonie Dejas, Juan Duque-Yate, Joël Boutin, Katherine Medina-Ortiz, Jacinthlyn Sylvia Suresh, Jordan Swiegers, Philippe Sarret, and Dimitri Ryczko

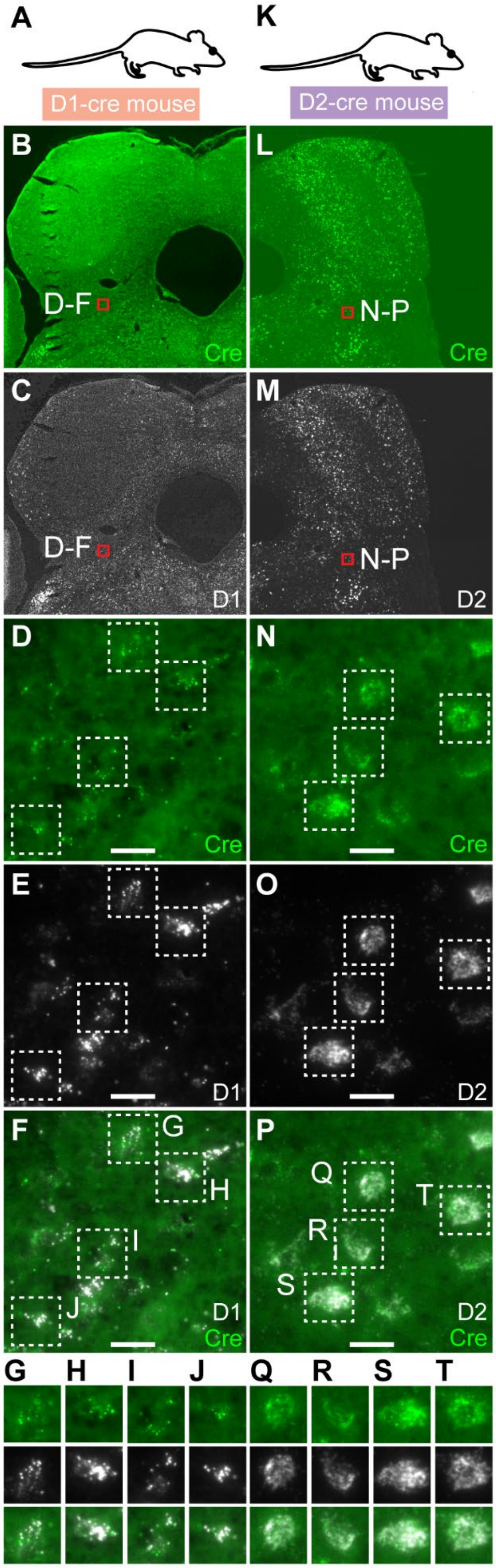

Figure S1. Expression of *cre mRNA* in D_1-cre and D_2-cre mice in the Mesencephalic Locomotor Region. Related to Figure 2. (A-F) Photomicrographs from a D_1-cre mouse illustrating example cells expressing both *cre mRNA* (green, B,D,F) and *D_1 receptor mRNA* (white, C,E,F). D-F illustrate magnifications of the region delineated in red in B-C. Cell count in 3 D_1-cre mice indicated that 86% of MLR neurons positive for *cre mRNA* expressed *D_1 receptor mRNA* (17/19 *cre mRNA*$^+$ cells in mouse 1, 26/32 in mouse 2, 25/29 in mouse 3, 1 to 3 slices per mouse). **(G-J)** Single cells extracted from the dashed squares in D-F. **(K-P)** Photomicrographs from a D_2-cre mouse illustrating example cells showing expression of *cre mRNA* (green, L,N,P) and *D_2 receptor mRNA* (white, M,O,P). N-P illustrate magnifications of the region delineated in red in L-M. Cell count in 3 D_2-cre mice indicated that 90% of MLR neurons positive for *cre mRNA* expressed *D_2 receptor mRNA* (17/17 *cre mRNA*$^+$ cells in mouse 1, 26/32 in mouse 2, 24/27 in mouse 3; 1 to 3 slices per mouse). **(Q-T)** Single cells extracted from the dashed squares in N-P. Scale bars, 20 μm.

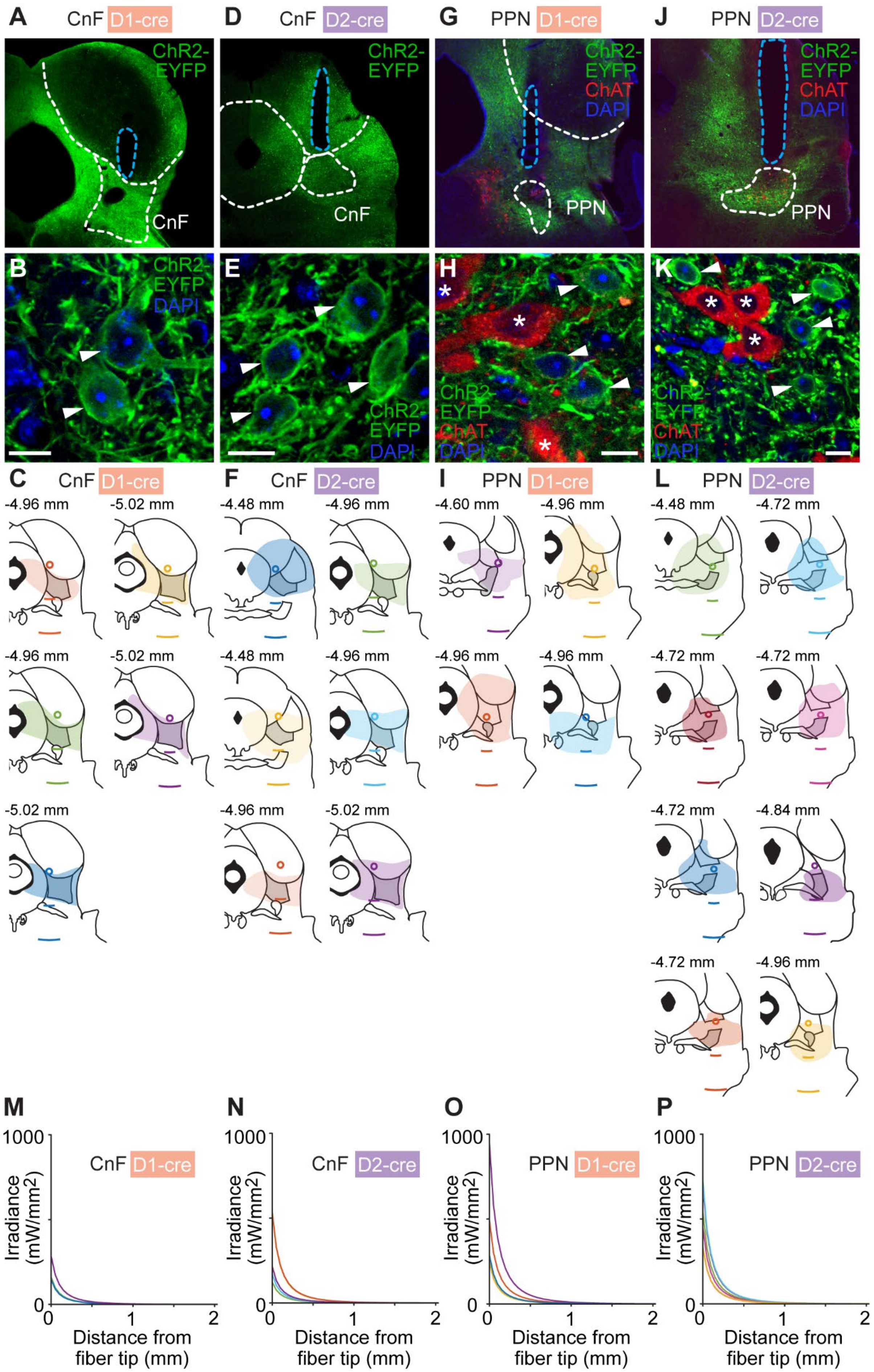

A
CnF D1-cre
ChR2-EYFP
CnF
D
CnF D2-cre
G
PPN D1-cre
ChR2-EYFP
ChAT
DAPI
PPN
J
PPN D2-cre
B
DAPI
E
H
K
C
-4.96 mm
-5.02 mm
F
-4.48 mm
I
-4.60 mm
L
-4.72 mm
-4.84 mm
M
N
O
P
Irradiance (mW/mm2)
1000
0
1
2
Distance from fiber tip (mm)

Figure S2. Adeno-associated virus injections and optic fiber implantations. Related to Figure 2. (A,D,G,J) Photomicrographs showing the position of cells infected by virus injection (green) in the cuneiform nucleus (CnF) or pedunculopontine nucleus (PPN) and position of the optic fiber right above the CnF (A,D) or PPN (G,J) in D_1-cre mice (A,G) or D_2-cre mice (D,J). In G and J, cells immunoreactive for choline acetyltransferase (ChAT) are shown in red, the nuclear marker DAPI is shown in blue. **(B,E,H,K)** Confocal magnifications of the CnF or PPN regions delineated in A,D,G,J, showing example cells (arrowheads) expressing channelrhodopsin fused with enhanced yellow fluorescent protein (ChR2-EYFP, green) driven by the adeno-associated virus in a cre-dependent manner. In H and K, cells immunoreactive for ChAT are shown in red (asterisks) and the nuclear marker DAPI is shown in blue. Scale bars, 10 µm. **(C,F,I,L)** Histological locations of the cells infected by the adeno-associated virus and of the tips of the optic fibers. On each slice, the 18.6° arcs illustrate the estimated cones of irradiance at 1 mm and 2 mm away from optic fiber tip for the maximal laser intensity used for each mouse. The cone apex angle was defined based on measurements done in brain tissue (see STAR Methods). **(M-P)** For each animal group, estimated decrease of irradiance as a function of distance from optic fiber tip based on measurements done in brain tissue (see STAR Methods). The colors for each animal are the same as in C,F,I,L. Note that in some mice the same maximal laser intensity was used, causing trace overlap here.

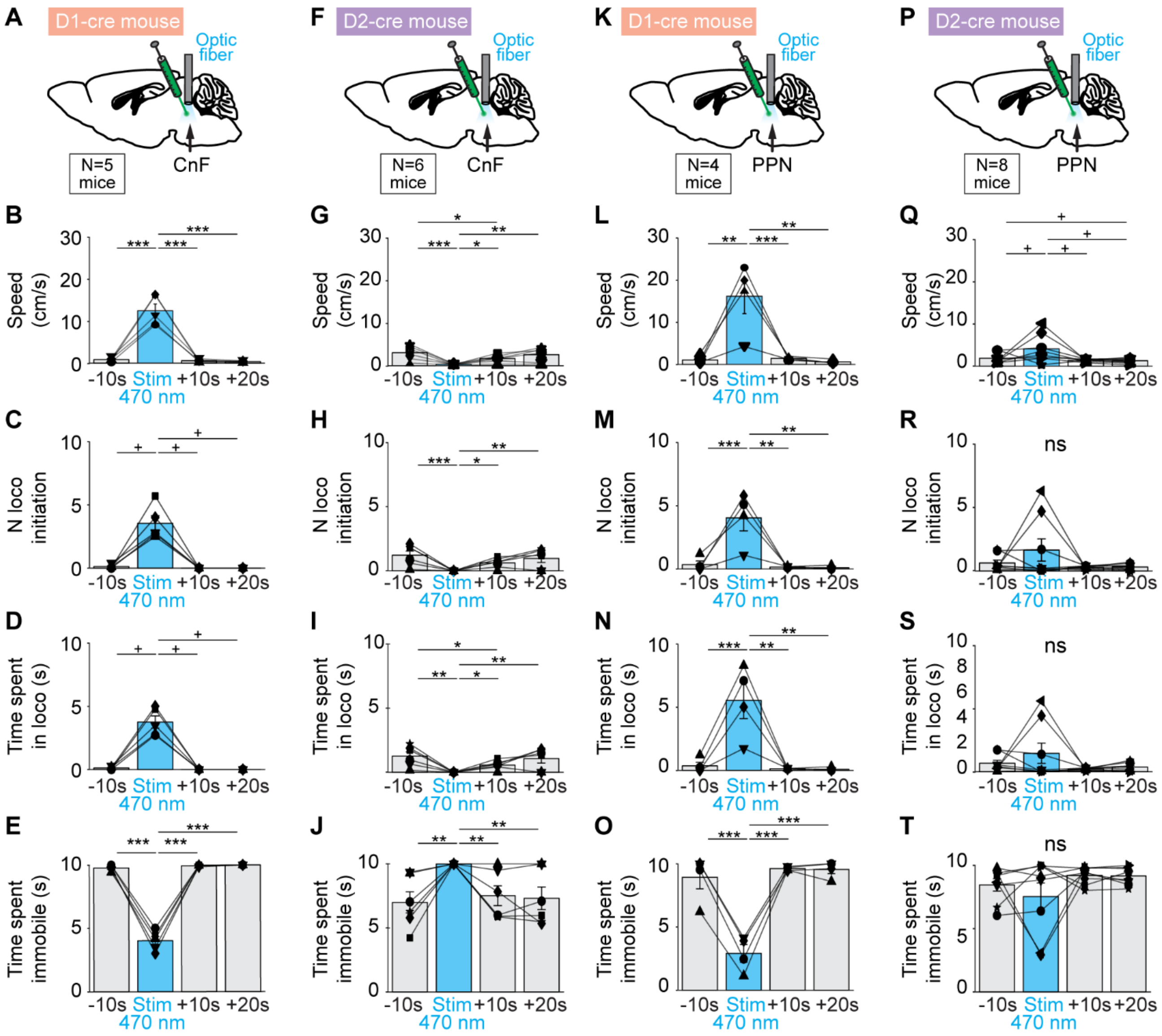

Figure S3. Effect of optogenetic activation with blue light (470 nm) of MLR dopamine-sensitive neurons on locomotor parameters. Related to Figure 2. (A-T) Evolution of locomotor parameters (mean ± sem) (see STAR Methods) before (-10 to 0 s), during (0 to 10 s), and after (10 to 20 s and 20 to 30 s) optogenetic stimulation with a blue laser (470 nm, 10 s train, 20 Hz, 10 ms pulses) of CnF in D_1-cre mice (9-15% of laser power) (A-E), CnF in D_2-cre mice (8-12% of laser power) (F-J), PPN in D_1-cre mice (10-24% of laser power) (K-O) and PPN in D_2-cre mice (10-31% of laser power) (P-T). *P<0.05, **P<0.01, ***P<0.001, Student–Newman–Keuls test after a one-way ANOVA for repeated measures (P<0.001 in B,E,G,H,N,O; P<0.01 in I,J,L,M; ns, not significant P>0.05 in T); +P<0.05, Student–Newman–Keuls test after a Friedman repeated measures ANOVA on ranks (P < 0.01 in C,D,Q); ns, not significant one-way ANOVA for repeated measures (P > 0.05 in R,S). When comparing CnF D_1, CnF D_2, PPN D_1 and PPN D_2 animal groups, locomotor speed during the 10 s before stimulation was not different between groups, except for the CnF D_2 group that displayed a higher speed than the CnF D_1 group (P < 0.05 Student Newman-Keuls after a One Way ANOVA, P < 0.05).

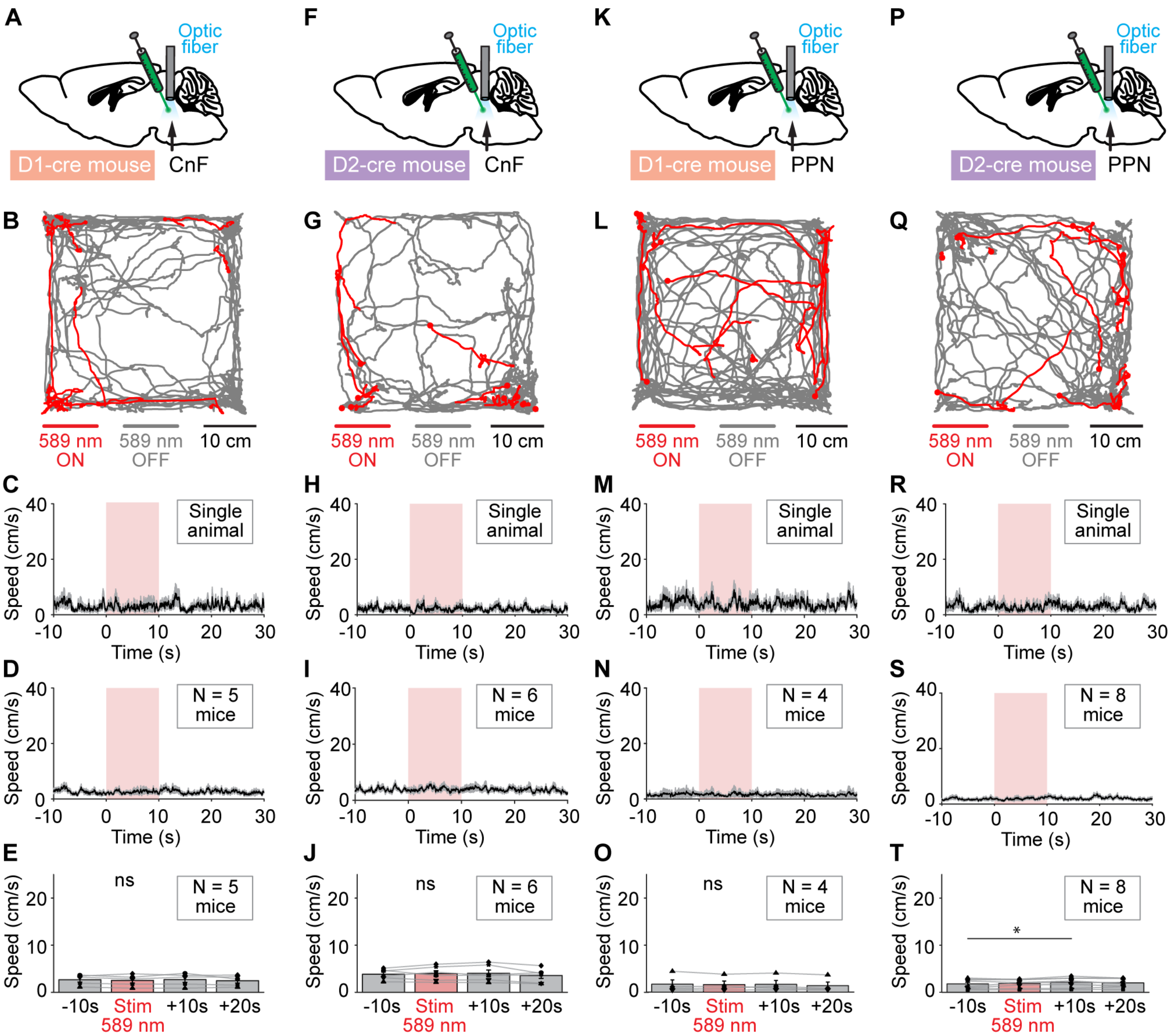

Figure S4. Effect of optogenetic activation with red light (589 nm) of MLR dopamine-sensitive neurons on locomotion. Related to Figure 2. (A-T) Locomotor activity and evolution of speed (mean ± sem) (see STAR Methods) before (-10 to 0 s), during (0 to 10 s), and after (10 to 20 s and 20 to 30 s) optogenetic stimulation with a red laser (589 nm, 10 s train, 20 Hz, 10 ms pulses, 55% of laser power) of CnF in D_1-cre mice (A-E), CnF in D_2-cre mice (F-J), PPN in D_1-cre mice (K-O) and PPN in D_2-cre mice (P-T). *$P<0.05$, Student–Newman–Keuls test after a one-way ANOVA for repeated measures ($P<0.05$ in T); ns, not significant one-way ANOVA for repeated measures ($P>0.05$ in E,J,O).

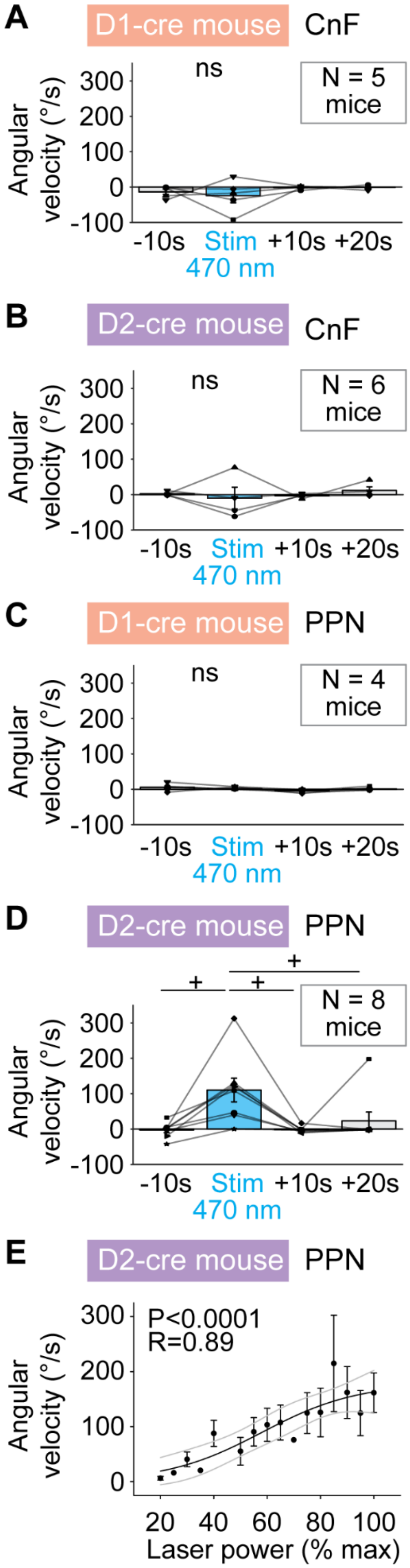

Figure S5. Effect of optogenetic activation with blue light (470 nm) of dopamine-sensitive neurons in the cuneiform nucleus (CnF) or pedunculopontine nucleus (PPN) on angular velocity. Related to Figure 3. (A-D) Evolution of angular velocity (mean ± sem) in the open field arena (see STAR Methods) before (-10 to 0 s), during (0 to 10 s), and after (10 to 20 s and 20 to 30 s) optogenetic stimulation with a blue laser (470 nm, 10 s train, 20 Hz, 10 ms pulses) of CnF in D_1-cre mice (9-15% of laser power) (A), CnF in D_2-cre mice (8-12% of laser power) (B), PPN in D_1-cre mice (10-24% of laser power) (C) and PPN in D_2-cre mice (10-31% of laser power) (D). $^+P<0.05$, Student–Newman–Keuls test after a Friedman repeated measures ANOVA on ranks ($P<0.05$); ns, not significant one-way ANOVA for repeated measures ($P>0.05$ in A-C). **(E)** Relationship between angular velocity (mean ± SEM) and laser power (470 nm, 10 s train, 20 Hz, 10 ms pulses, 4-34% of laser power, bin width 5%, 1 to 8 mice per bin) applied in the PPN of D_2-cre mice. The data followed a sigmoidal function (solid black line). The coefficient of correlation (R), its significance (P), and the confidence intervals (gray) are illustrated.

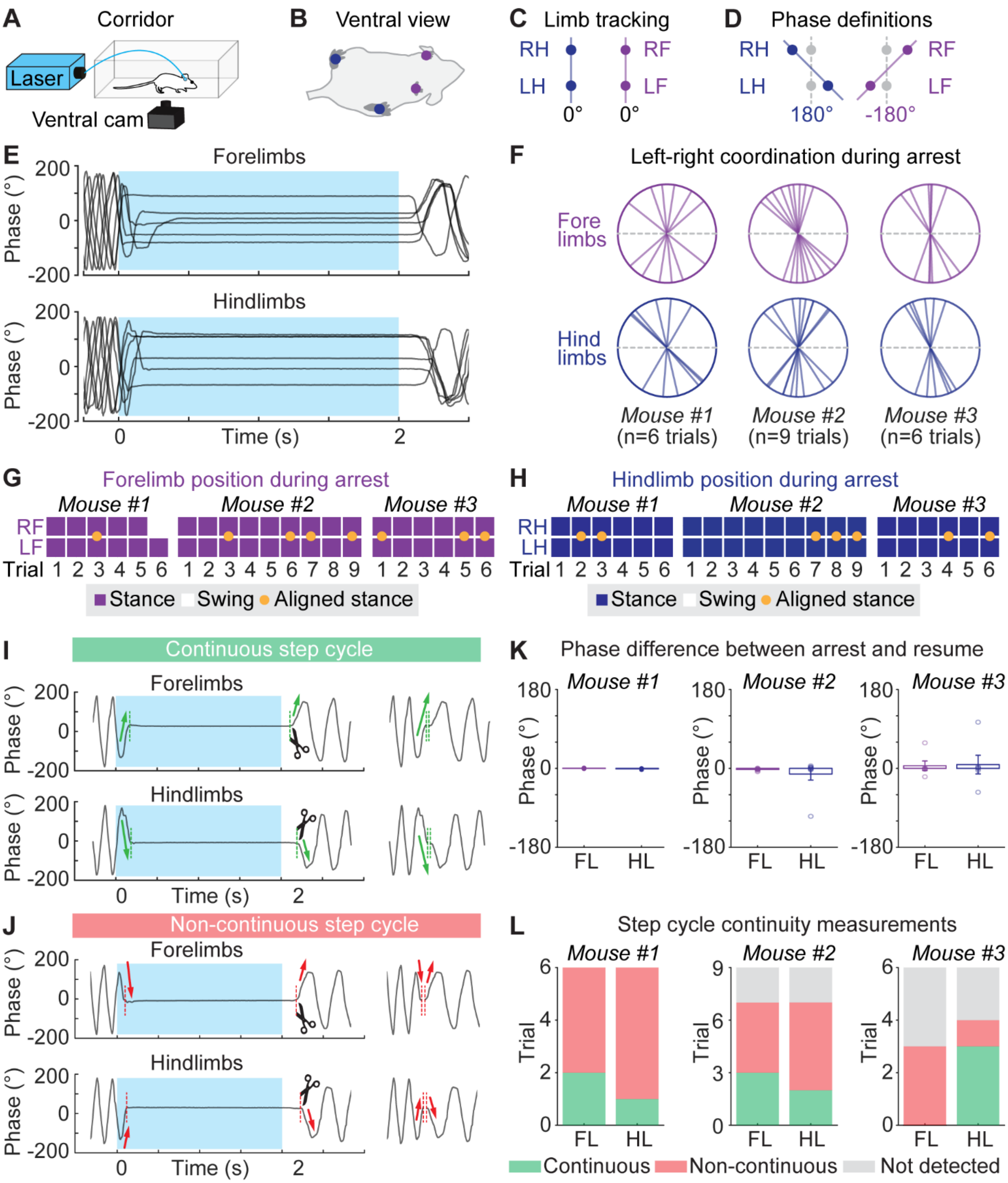

Figure S6. Coordination of limb movements during arrests evoked by CnF photostimulation in D2-cre mice. Related to Figure 5. (A) Limb movements were filmed from below in a transparent linear corridor (see STAR Methods). **(B)** Forelimb and hindlimb paws were detected using DeepLabCut (see STAR Methods). **(C-D)** To analyze left-right forelimb coordination based on paw tracking, phase values were defined by the distance on the x axis (i.e. corridor length axis) between left and right forelimbs. The same analysis was carried out for hindlimbs. **(E)** Left-right coordination phase values for forelimbs (top) and hindlimbs (bottom) during locomotion temporarily interrupted by CnF photostimulation in an example D2-cre mouse (n = 6 trials) (470 nm, 2 s train, 20 Hz, 10 ms pulses, 10% of laser power). **(F)** Left-right coordination for forelimbs (top) and hindlimbs (bottom) during arrest evoked CnF photostimulation in D2-cre mice (n = 3 mice, 6-9 trials per mouse, 470 nm, 2 s train, 20 Hz, 10 ms pulses, 10-12% of laser power). For each trial, the line that unites the left and right forelimb paws, or

the left and right hindlimb paws is drawn in a circle. Dashed grey lines correspond to the corridor length axis. **(G-H)** Forelimb (purple) and hindlimb (blue) positions during arrest in the corridor for CnF stimulation trials in D_2-cre mice (n = 3 mice, 6-9 trials per mouse). Solid squares represent stance, and empty squares represent swing. Yellow dots illustrate that RH and LH, or RF and LF were aligned perpendicular (90 ± 15º) to the corridor length axis. **(I)** Representative trial illustrating continuity in the step cycle after light offset. **(J)** Representative trial illustrating non-continuity in the step cycle after light offset. **(K)** Phase difference (mean ± sem) between arrest time points and resume time points for forelimbs (FL) and hindlimbs (HL) in three mice (6-9 trials per mouse). **(L)** Step cycle continuities and non-continuities illustrated by arrows in I-J, quantified as a binary outcome for all trials for each of the three mice tested. Circles represent individual trials (n = 3 mice, 6-9 trials per mouse). Blue rectangles (E, I and J) illustrate photostimulation duration.

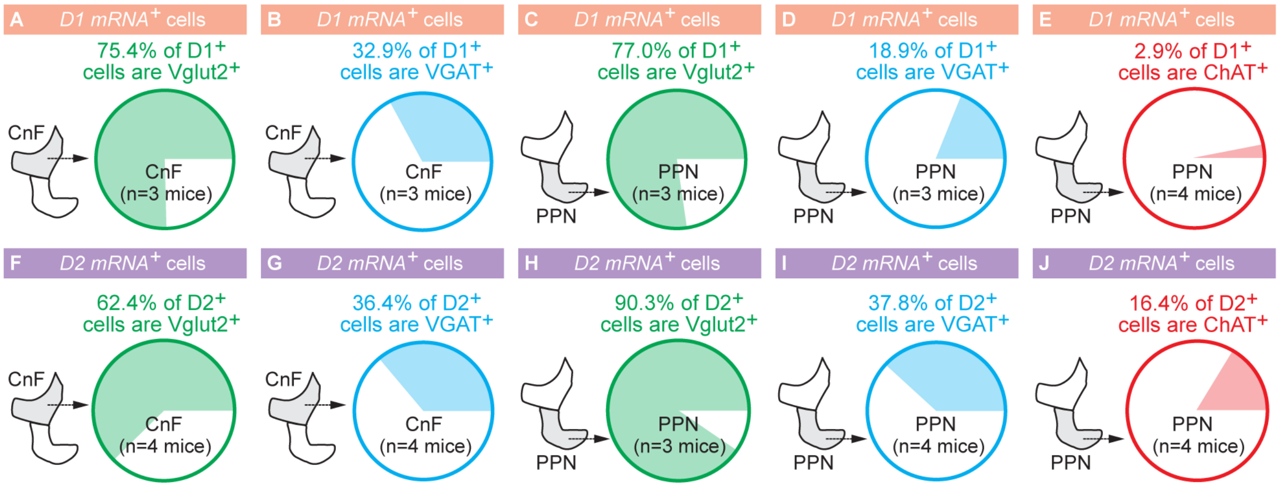

Figure S7. Proportions of *Vglut2 mRNA*$^+$, *VGAT mRNA*$^+$ and *ChAT mRNA*$^+$ neurons among D_1 or D_2 receptor-expressing neurons in the cuneiform nucleus (CnF) or pedunculopontine nucleus (PPN). Related to Figure 7. (A-B) Proportions of *D_1 receptor mRNA*$^+$ cells in the CnF expressing *Vglut2 mRNA* (A, 18/21 *D_1 receptor mRNA*$^+$ cells in mouse 1, 58/70 in mouse 2, 36/63 in mouse 3) or *VGAT mRNA* (B, 13/29 *D_1 receptor mRNA*$^+$ cells in mouse 1, 14/51 in mouse 2, 10/31 in mouse 3). **(C-E)** Proportions of *D_1 receptor mRNA*$^+$ cells in the PPN expressing *Vglut2 mRNA* (C, 31/48 *D_1 receptor mRNA*$^+$ cells in mouse 1, 54/64 in mouse 2, 58/70 in mouse 3) or *VGAT mRNA* (D, 3/33 *D_1 receptor mRNA*$^+$ cells in mouse 1, 12/49 in mouse 2, 9/38 in mouse 3) or *ChAT mRNA* (E, 0/17 *D_1 receptor mRNA*$^+$ cells in mouse 1, 1/22 in mouse 2, 1/34 in mouse 3, 2/45 in mouse 4). **(F-G)** Proportions of *D_2 receptor mRNA*$^+$ cells in the CnF expressing *Vglut2 mRNA* (F, 20/48 *D_2 receptor mRNA*$^+$ cells in mouse 1, 47/65 in mouse 2, 23/38 in mouse 3, 15/20 in mouse 4) or *VGAT mRNA* (G, 15/51 *D_2 receptor mRNA*$^+$ cells in mouse 1, 35/113 in mouse 2, 23/56 in mouse 3, 33/81 in mouse 4). **(H-J)** Proportions of *D_2 receptor mRNA*$^+$ cells in the PPN expressing *Vglut2 mRNA* (H, 58/61 *D_2 receptor mRNA*$^+$ cells in mouse 1, 25/30 in mouse 2, 12/13 in mouse 3) or *VGAT mRNA* (I, 21/35 *D_2 receptor mRNA*$^+$ cells in mouse 1, 28/83 in mouse 2, 15/41 in mouse 3, 18/84 in mouse 4) or ChAT mRNA (J, 3/36 *D_2 receptor mRNA*$^+$ cells in mouse 1, 8/61 in mouse 2, 17/87 in mouse 3, 18/79 in mouse 4). In A-J, quantifications were made from 1 to 3 slices per mouse.

MLR cell populations expressing D1 or D2 receptors control distinct aspects of behavior

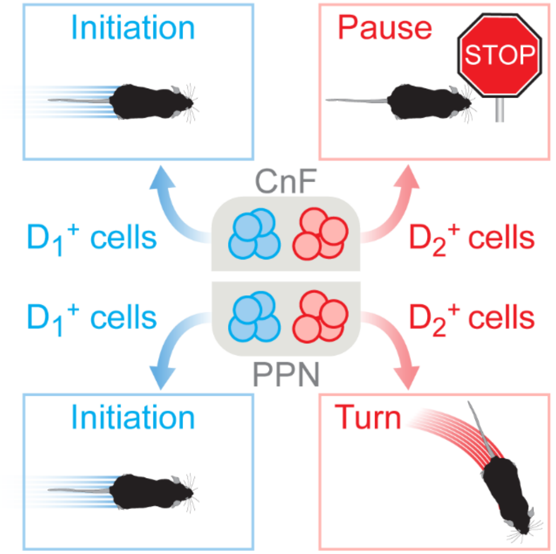

Figure S8. Behavioral roles of the dopamine-sensitive neurons in the mammalian Mesencephalic Locomotor Region (MLR). Related to Figure 2.
