# Supplementary material for: Dopamine-sensitive neurons in the mesencephalic locomotor region control locomotion initiation, stop, and turns
Source: Cell Rep. 2024 May 8;43(5):114187. doi: 10.1016/j.celrep.2024.114187 (PMC11157412; doi:10.1016/j.celrep.2024.114187)
Supplement: Document S2. Article plus supplemental information [file mmc10.pdf]

Cell Reports

Article

Dopamine-sensitive neurons in the mesencephalic locomotor region control locomotion initiation, stop, and turns

Graphical abstract

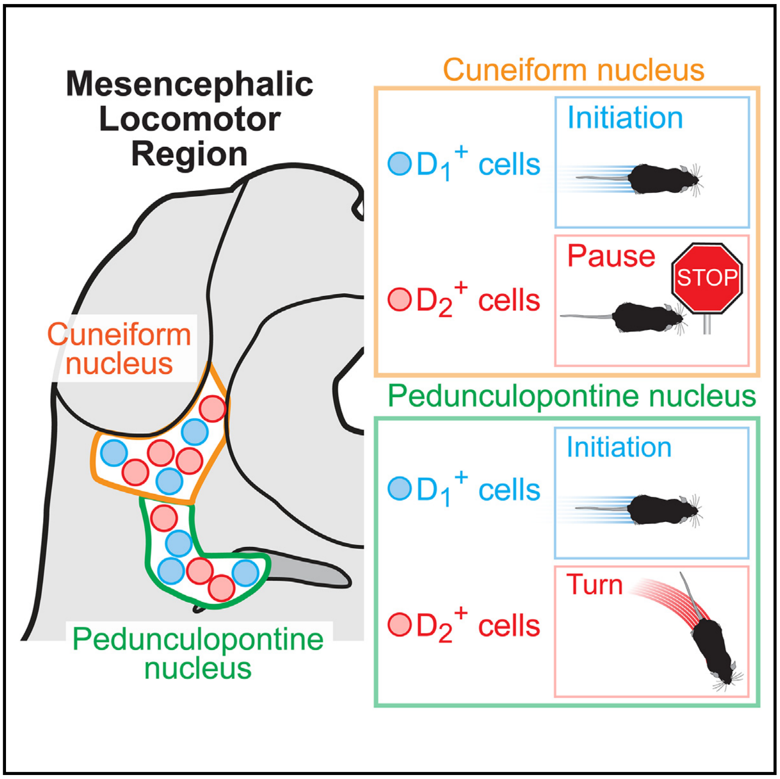

Highlights

- Activation of D_1^+ cells in the cuneiform or pedunculopontine nuclei evokes locomotion
- Activation of D_2^+ cells in the cuneiform nucleus stops locomotion
- Activation of D_2^+ cells in the pedunculopontine nucleus induces ipsilateral turning
- Glutamatergic, GABAergic, and cholinergic MLR cells express dopaminergic receptors

Authors

Andrea Juárez Tello, Cornelis Immanuel van der Zouwen, Léonie Dejas, ..., Jordan Swiegers, Philippe Sarret, Dimitri Ryczko

Correspondence

dimitri.ryczko@gmail.com

In brief

Juárez Tello et al. show that dopamine-sensitive neurons in the mesencephalic locomotor region control movement. D_1 receptor-positive neurons in the cuneiform and pedunculopontine nuclei promote locomotion. D_2 receptor-positive neurons in the cuneiform nucleus stop locomotion, while D_2 receptor-positive neurons in the pedunculopontine nucleus induce ipsilateral turning.

Juárez Tello et al., 2024, Cell Reports *43*, 114187
May 28, 2024 © 2024 The Author(s). Published by Elsevier Inc.
https://doi.org/10.1016/j.celrep.2024.114187

CellPress

Cell Reports

CellPress
OPEN ACCESS

Article

Dopamine-sensitive neurons in the mesencephalic locomotor region control locomotion initiation, stop, and turns

Andrea Juárez Tello,[1,4] Cornelis Immanuel van der Zouwen,[1,4] Léonie Dejas,[1,4] Juan Duque-Yate,[1] Joël Boutin,[1] Katherine Medina-Ortiz,[1] Jacinthlyn Sylvia Suresh,[1] Jordan Swiegers,[1] Philippe Sarret,[1,2,3] and Dimitri Ryczko[1,2,3,5,*]
[1]Département de Pharmacologie-Physiologie, Faculté de médecine et des sciences de la santé, Université de Sherbrooke, Sherbrooke, QC, Canada
[2]Centre de recherche du Centre Hospitalier Universitaire de Sherbrooke, Sherbrooke, QC, Canada
[3]Neurosciences Sherbrooke, Institut de Pharmacologie de Sherbrooke, Sherbrooke, QC, Canada
[4]These authors contributed equally
[5]Lead contact
*Correspondence: dimitri.ryczko@gmail.com
https://doi.org/10.1016/j.celrep.2024.114187

SUMMARY

The locomotor role of dopaminergic neurons is traditionally attributed to their ascending projections to the basal ganglia, which project to the mesencephalic locomotor region (MLR). In addition, descending dopaminergic projections to the MLR are present from basal vertebrates to mammals. However, the neurons targeted in the MLR and their behavioral role are unknown in mammals. Here, we identify genetically defined MLR cells that express D_1 or D_2 receptors and control different motor behaviors in mice. In the cuneiform nucleus, D_1-expressing neurons promote locomotion, while D_2-expressing neurons stop locomotion. In the pedunculopontine nucleus, D_1-expressing neurons promote locomotion, while D_2-expressing neurons evoke ipsilateral turns. Using RNAscope, we show that MLR dopamine-sensitive neurons comprise a combination of glutamatergic, GABAergic, and cholinergic neurons, suggesting that different neurotransmitter-based cell types work together to control distinct behavioral modules. Altogether, our study uncovers behaviorally relevant cell types in the mammalian MLR based on the expression of dopaminergic receptors.

INTRODUCTION

Locomotion is an essential daily activity. Depending on their needs, animals may initiate locomotion to explore their environment, stop carefully to approach prey or avoid detection by a predator, or turn to move around an obstacle. The major command systems for these motor actions are located in the brainstem. A key structure is the mesencephalic locomotor region (MLR), which plays an important role in controlling locomotor initiation, speed, and gait transitions (see recent reviews[1–4]).

The MLR is traditionally considered to be indirectly modulated by the dopaminergic system. Dopaminergic cells of the substantia nigra pars compacta send ascending projections to the striatum, the entry station of the basal ganglia whose output structures inhibit MLR glutamatergic neurons.[5] Dopaminergic activity in the striatum precedes the initiation of locomotion and invigorates future movements.[6–8] This is mediated by two populations of dopamine-sensitive neurons in the striatum. Striatal neurons expressing D_1 receptors promote locomotion by decreasing the inhibitory activity sent from the basal ganglia output stations to the MLR, whereas neurons expressing D_2 receptors have the opposite effect.[9]

In addition to this well-established circuitry, the MLR was recently found to receive direct descending projections from meso-diencephalic dopaminergic neurons from basal vertebrates to mammals[10–14] (for review, see Ryczko and Dubuc[15]). Stimulation of the meso-diencephalic dopaminergic region evokes dopamine release in the MLR in lamprey, salamander, and rat, and amphetamine increases dopamine release *in vivo* in rat.[10,11] However, in the mammalian MLR, little is known about the targets of dopaminergic inputs, the expression of dopaminergic receptors, and the behavioral role of dopamine-sensitive neurons.

The mammalian MLR comprises dorsally the cuneiform nucleus (CnF) and ventrally the pedunculopontine nucleus (PPN). These regions comprise a combination of glutamatergic, GABAergic, and cholinergic neurons that control distinct aspects of locomotion (see recent reviews[1–4]). However, in mammals, whether these different cell types express dopaminergic receptors is unknown, and the behavioral role of MLR dopamine-sensitive cells is unknown. In lamprey, pharmacological blockade of D_1 receptors in the MLR decreases locomotor activity evoked by stimulation of the meso-diencephalic dopaminergic region,[10,12] suggesting that at least D_1 receptors may be expressed in the mammalian MLR.

This is an open access article under the CC BY license (http://creativecommons.org/licenses/by/4.0/).

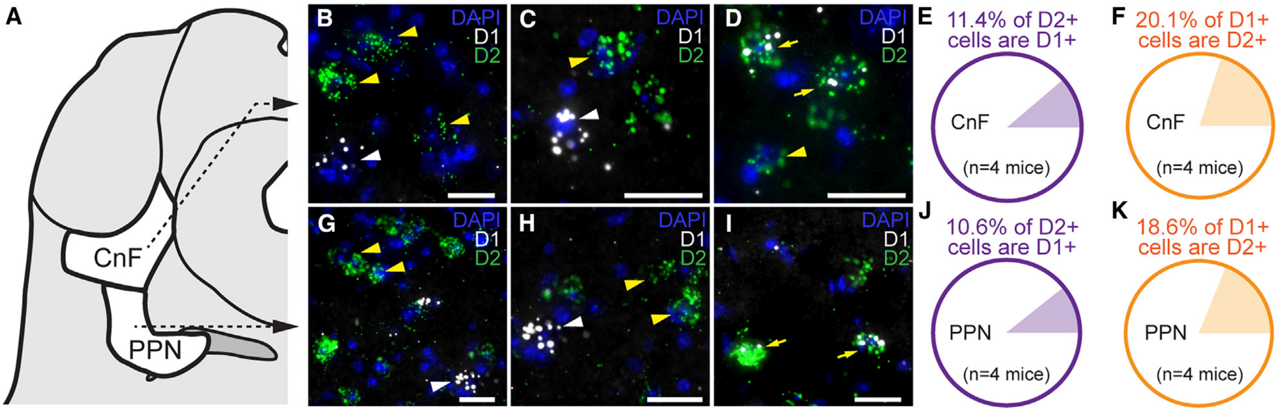

Figure 1. Expression of dopaminergic D_1 mRNA and D_2 mRNA in neurons of the mesencephalic locomotor region

(A) Scheme illustrating the CnF and PPN.

(B–D) and (G–I) Example photomicrographs illustrating the expression in wild-type mice of D_1 receptor mRNA (white) and D_2 receptor mRNA (green) in the CnF (B–D) or PPN (G–I). The nuclear marker DAPI appears in blue. Yellow arrowheads illustrate example cells only positive for D_2 receptor mRNA, and white arrowhead illustrates example cells only positive for D_1 mRNA. Yellow arrows illustrate example cells positive both for D_1 receptor mRNA and D_2 receptor mRNA.

(E and F) and (J and K) Proportions of cells expressing D_2 receptor mRNA that co-express D_1 receptor mRNA in the CnF (E, 2/22 D_2^+ cells in mouse 1, 5/41 in mouse 2, 2/27 in mouse 3, 11/75 in mouse 4) or PPN (J, 1/12 D_2^+ cells in mouse 1, 10/50 in mouse 2, 1/33 in mouse 3, 8/75 in mouse 4), and proportions of cells expressing D_1 mRNA that co-express D_2 mRNA in the CnF (F, 2/8 D_1^+ cells in mouse 1, 5/45 in mouse 2, 2/10 in mouse 3, 11/48 in mouse 4) and PPN (K, 1/8 D_1^+ cells in mouse 1, 10/35 in mouse 2, 1/15 in mouse 3, 8/35 in mouse 4). In (E and F) and (J and K) quantifications made from 1 to 3 slices per mouse. Scale bars, 25 μm.

Here, we have identified genetically defined populations of dopamine-sensitive neurons expressing D_1 or D_2 receptors in the CnF and PPN. Using optogenetics coupled with deep learning-based movement analysis in freely moving mice, we show that, in the CnF, activation of D_1-expressing neurons evokes locomotion, while activation of D_2-expressing neurons stops locomotion. In the PPN, activation of D_1-expressing neurons promotes locomotion, while activation of D_2-expressing neurons evokes ipsilateral turns. We found that MLR dopamine-sensitive neurons contain a diversity of glutamatergic, GABAergic, and cholinergic neurons, which probably act synergistically to produce behavior. Our study shows that the expression of D_1 or D_2 receptors in the MLR defines behaviorally relevant cell populations. These results provide an additional dopaminergic substrate through which dopamine can influence locomotion in mammals. Such circuitry is in a good position to contribute to the locomotor effects of psychostimulants. Loss of dopaminergic inputs to these dopaminoceptive neurons may result in locomotor deficits in Parkinson's disease.

RESULTS

MLR neurons express D_1 and D_2 receptor mRNAs

Using RNAscope in mouse brain slices, we found that D_1 and D_2 receptor mRNAs are expressed in the CnF and PPN (Figures 1A–1K). Cells expressing D_1 or D_2 receptor mRNA mostly constituted segregated populations. Only 11.4% of CnF cells and 10.6% of PPN cells positive for D_2 mRNA were positive for D_1 mRNA. Likewise, 20.1% of CnF cells and 18.6% of PPN cells positive for D_1 mRNA were positive for D_2 mRNA (Figures 1A–1K, n = 4 mice).

D_1-positive CnF neurons promote locomotion

We investigated the behavioral role of MLR neurons expressing D_1 receptors using *in vivo* optogenetics in D_1-cre mice[16,17] (see STAR Methods and Figures S1A–S1J). To activate neurons expressing D_1 receptors, we injected in the CnF of D_1-cre mice an adeno-associated virus (AAV) driving expression of the light-sensitive cation channel channelrhodopsin in a cre-dependent manner (see STAR Methods) and implanted an optic fiber 500 μm above the injection site (Figures S2A–S2C and S2M). Blue light (470 nm) photostimulation trains applied to the CnF in D_1-cre mice increased locomotor speed in the open-field arena (Video S1). Statistical analyses revealed that the stimulation increased the number of locomotion initiations, increased the time spent in locomotion, and reduced the time spent immobile (Figures 2A–2C and S3A–S3E, n = 5 mice). No effect on locomotor speed was seen when 470 nm light was replaced with 589 nm light (Figures S4A–S4E, n = 5 mice). A major function of the CnF is to control locomotor speed.[18–21] In D_1-cre mice, increasing the laser power applied during CnF photostimulation increased locomotor speed (Figure 2D). We found a sigmoidal relationship between laser power applied to CnF and locomotor speed in D_1-cre mice (R = 0.91, $p < 0.001$, n = 5 mice; Figure 2E).

CnF photostimulation in D_1-cre mice mainly evoked straight forward locomotion in the open-field arena with turns occurring when approaching a wall (Figure 2B; Video S1). This is illustrated by the body orientation vectors, drawn between tail base position and body center position (Figures 3A, 3E, and 3F), and by the body trajectory vectors, drawn between body center positions in consecutive frames (Figures 3J and 3K). The average angular velocity of the animal's trajectory was centered around zero during optogenetic stimulation, and there was no difference

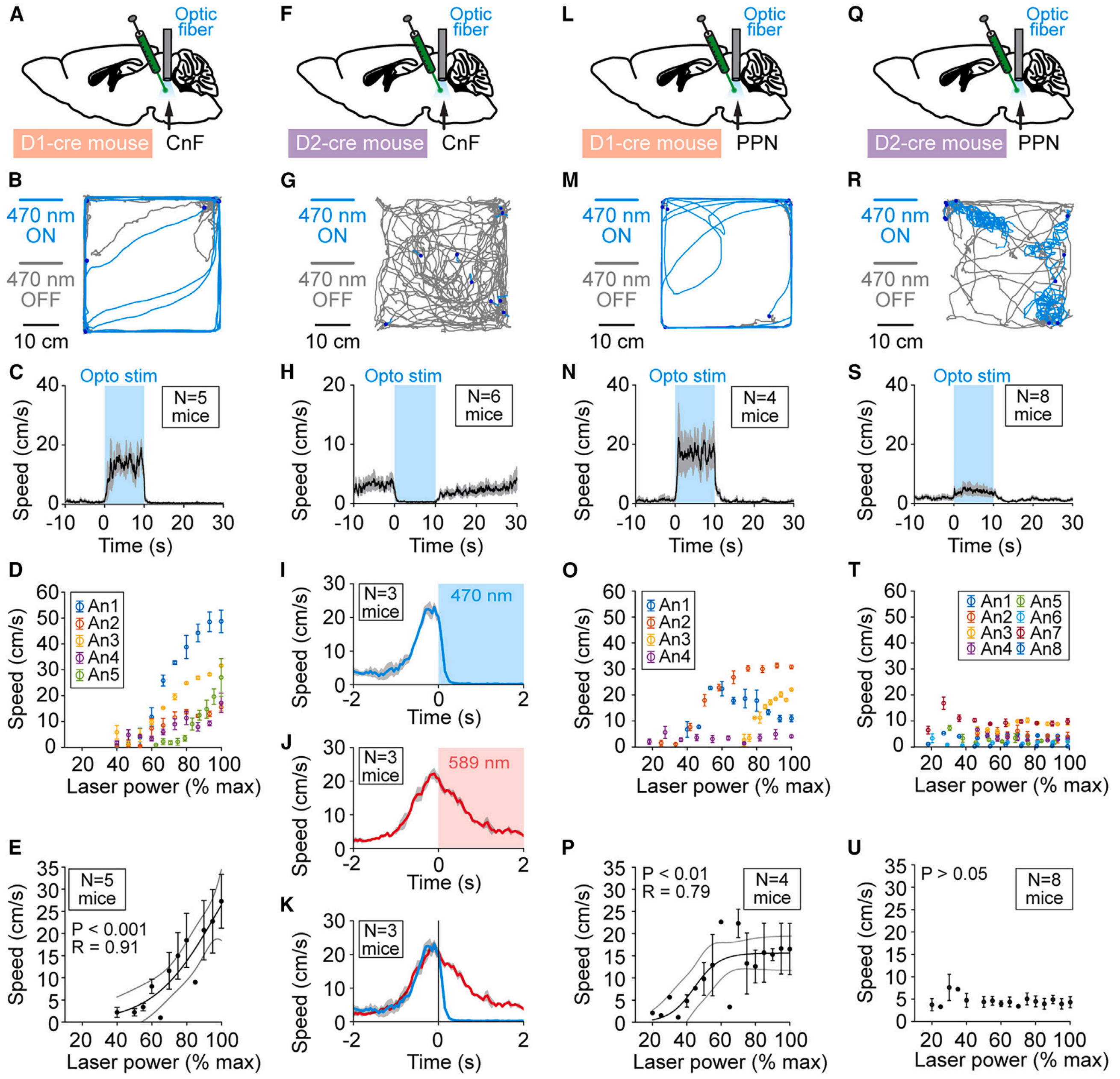

Figure 2. D_1- and D_2 receptor-expressing MLR neurons control distinct aspect of movement

(A, F, L, and Q) D_1-cre or D_2-cre mice were injected in the CnF or PPN with an adeno-associated virus encoding for channelrhodopsin in a cre-dependent manner (see STAR Methods) and implanted with an optic fiber ~500 μm above the injection site.

(B, G, M, and R) Mouse trajectories in the open field during spontaneous locomotion (gray lines) and during multiple 10-s optogenetic stimulations (blue lines; dots indicate start of stimulation) in D_1-cre mice or D_2-cre mice in CnF or PPN with a 470-nm laser (10-s train, 20 Hz, 10-ms pulses; B, 15%; G, 10%; M, 13.2%; R, 12% of laser power).

(C, H, N, and S) Locomotor speed (mean ± SEM) before, during, and after a 10-s optogenetic stimulation (onset at t = 0 s; 10-s train, 20 Hz, 10-ms pulses; C, 9%–15%; H, 8%–12%; N, 10%–24%; S, 10%–31% of laser power).

(D, O, and T) Locomotor speed as a function of laser power (D, 6%–15%; O, 6%–44%; T, 4%–34% of laser power). Each dot represents speed (mean ± SEM) measured during one to three trials. Laser power was normalized as a function of their maximal value (bin size 5%).

(E, P, and U) Relationships between locomotor speed (mean ± SEM) and laser power (bin width 5%, 1 to 5 mice per bin) in the same mice shown in (D), (O), and (T). The data followed a sigmoidal function (solid black line) for CnF stimulation in D_1-cre mice (E), PPN stimulation in D_1-cre mice (P), but not for PPN stimulation in D_2-cre (U). When the fit is significant, the coefficient of correlation (R), its significance (P), and the confidence intervals (gray lines) are illustrated.

(I–K) Locomotor speed (mean ± SEM) before, during, and after a 2-s optogenetic stimulation (onset at t = 0 s) in CnF of D_2-cre mice with a 470-nm laser (20 Hz, 10-ms pulses, 8%–12% of laser power) or with a 589-nm laser (20 Hz, 10-ms pulses, 55% of laser power) in three mice. In (K) merged traces from (I) and (J). See also Figures S1–S4 and S8.

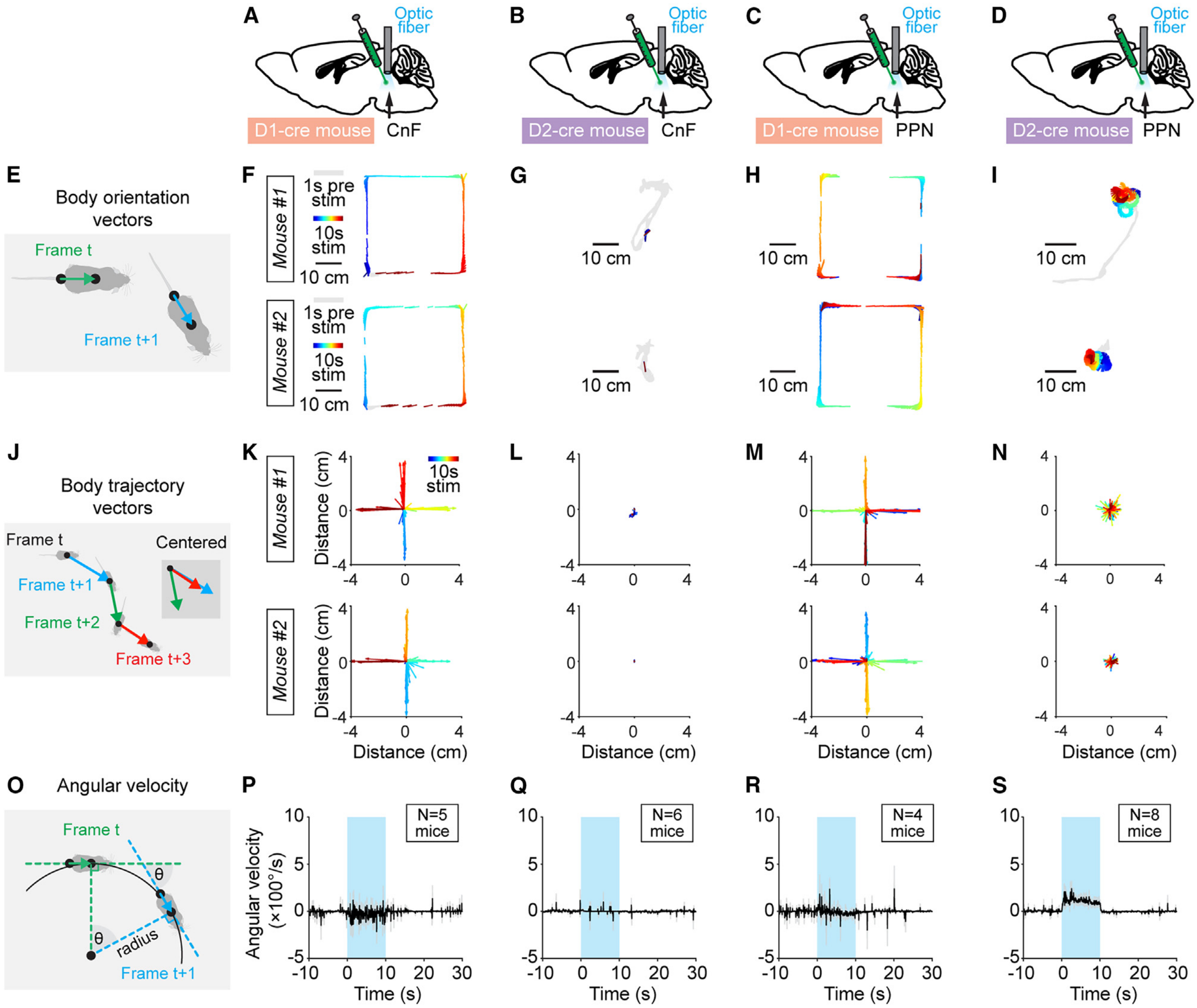

Figure 3. Directionality of locomotion is controlled by dopamine-sensitive MLR neurons

(A–D) D_1-cre or D_2-cre mice were injected in the CnF or PPN with an adeno-associated virus encoding for channelrhodopsin in a cre-dependent manner (see STAR Methods) and implanted with an optic fiber ~500 μm above injection site (same mice as in Figure 2, see Figure S2).

(E–I) Body orientation vectors in the open field extracted from two example mice as a function of time (color coded, warm colors illustrate the end of the recording). In (E), scheme illustrating how body orientation vectors were drawn frame by frame, from tail base to body center. Frames were excluded if the detection likelihood of body center or tail base positions was <0.8 (see STAR Methods).

(J–N) Body trajectory vectors extracted from two example mice as a function of time (color coded, warm colors illustrate the end of the recording). Vectors were centered on their starting positions. In (J), scheme illustrating how body trajectory vectors were drawn using consecutive frames, from body center position on frame t to body center position on frame t + 1 (see STAR Methods).

(O–S) Angular velocity (mean ± SEM) before, during, and after optogenetic stimulation (onset at t = 0 s) in D_1-cre mice or D_2-cre mice in CnF or PPN with a 470-nm laser (10-s train, 20 Hz, 10-ms pulses; P, 9%–15%; Q, 8%–12%; R, 10%–24%; S, 10%–31% of laser power). In (O), scheme illustrating how the angular velocity was calculated from frame to frame, using two lines drawn between tail base and body center at frame t and frame t + 1, and a circle that is tangential to both lines at the level of the two body center positions (see STAR Methods). See also Figure S5.

in angular velocity during stimulation compared with before or after stimulation, indicating that mice were moving mainly straight forward (Figures 3O, 3P, and S5A, n = 5 mice, see STAR Methods).

Hindlimb movements during locomotion evoked by CnF photostimulation in D_1-cre mice were largely normal. Joint angular excursions during optogenetic-evoked locomotion were similar to those recorded during spontaneous locomotion (p > 0.05, paired t test or Wilcoxon test, Figures 4A–4D, n = 4 mice, see STAR Methods). Altogether, this indicates that CnF D_1-positive neurons control locomotor initiation, locomotor speed, evoke largely normal hindlimb movements, and induce forward locomotion without impairing the animal's ability to turn when needed, i.e., when approaching a wall.

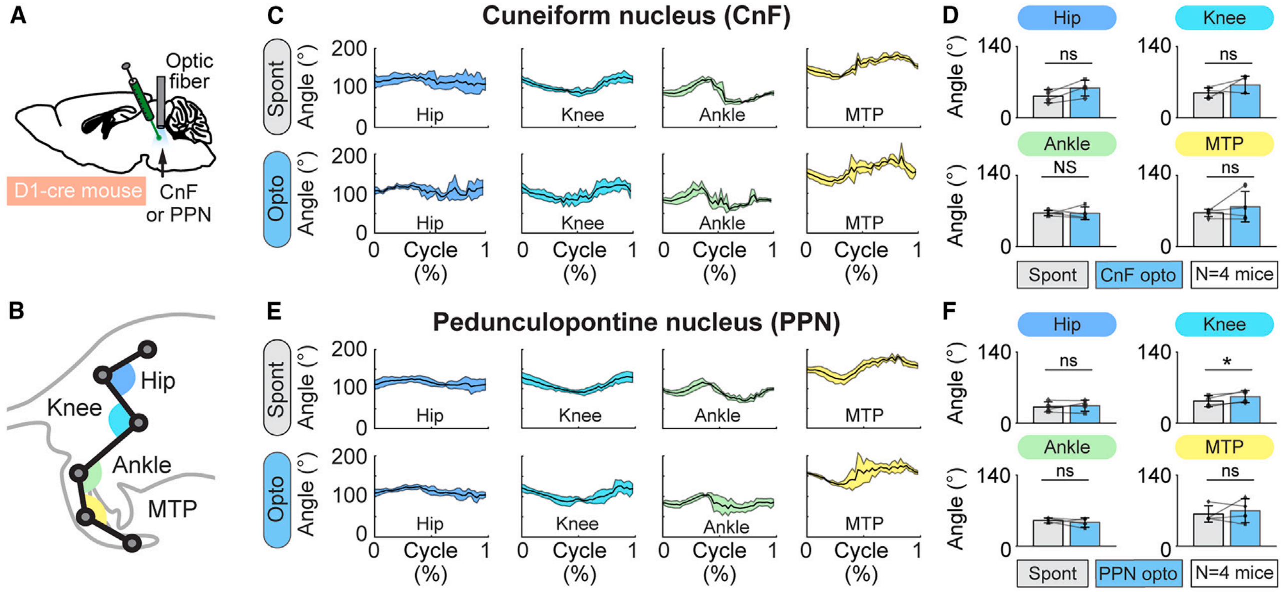

Figure 4. Hindlimb kinematics evoked by optogenetic stimulation of the CnF or PPN in D_1-cre mice

(A) D_1-cre mice were injected in CnF or PPN with an adeno-associated virus encoding for channelrhodopsin in a cre-dependent manner (see STAR Methods) and implanted with an optic fiber ~500 μm above the injection site (same mice as in Figure 2, see Figure S2).

(B) The movements of hindlimb joints were tracked from the side at 300 fps in a linear corridor during spontaneous locomotion (Spont) and during optogenetic-evoked locomotion with a 470-nm laser (10-s train, 20 Hz, 10-ms pulses) (Opto). The angles of the hip, knee, ankle, and metatarsophalangeal (MTP) joints were calculated frame by frame.

(C and E) Joint angles (mean ± SD) were plotted for a normalized locomotor cycle during spontaneous locomotion (top C, 9–23 steps per animal and top E, 12–41 steps per animal) and during optogenetic-evoked locomotion (bottom C, 4–19 steps and bottom E, 7–27 steps per animal, 8%–15% of laser power in C, 10%–28% of laser power in E). The cycle was defined as the time duration between two consecutive touchdowns of the MTP (see STAR Methods).

(D and F) Comparison of the amplitude of the hip, knee, ankle, and MTP angles (mean ± SD) during spontaneous locomotion and during locomotion evoked by optogenetic stimulation of the CnF (D, $n = 4$ mice) or PPN (F, $n = 4$ mice) in D_1-cre mice. ns, not significant, $p > 0.05$, *$p < 0.05$, paired t tests; NS, not significant, Wilcoxon test.

D_2-positive CnF neurons stop locomotion

We injected the same AAV encoding for channelrhodopsin expression in a cre-dependent manner into the CnF of D_2-cre mice[16,17] (see STAR Methods and Figures S1K–S1T) and implanted an optic fiber above the injection site (Figures S2D–S2F and S2N). In contrast with the results obtained in D_1-cre mice, CnF photostimulation in D_2-cre mice with blue light abruptly stopped ongoing movements in the open-field arena (Video S2). Photostimulation decreased the locomotor speed, decreased the number of locomotor initiations, decreased the time spent in locomotion, and increased the time spent immobile (Figures 2F–2H and S3F–S3J, $n = 6$ mice). No effect on locomotor speed was observed when 470 nm light was replaced with 589 nm light (Figures S4F–S4J, $n = 6$ mice).

Examination of body orientation vectors, body trajectory vectors, and angular velocity in the open-field arena showed that mice were largely immobile during the 10 s of CnF photostimulation in D_2-cre mice (Figures 3B, 3G, 3L, 3Q, and S5B). During ongoing locomotion in the open field, CnF photostimulation in D_2-cre mice decreased locomotor speed during the 2 s of stimulation with 470 nm light, compared with the control stimulation with 589 nm light (respectively, 1.7 ± 0.1 vs. 9.7 ± 0.3 cm/s, $p < 0.001$, paired t test, $n = 3$ mice, Figures 2I–2K). Such robust stops were also evident during ongoing locomotion in a linear corridor (Video S3), and when mice were walking on a treadmill (Video S4).

We examined body orientation and head rotation angles during such stops using an analysis inspired by Usseglio et al.[22] who reported locomotor stops when stimulating a subpopulation of $Chx10^+$ neurons in the gigantocellularis nucleus (see STAR Methods). CnF photostimulation in D_2-cre mice for 10 s did not modify body orientation angle ($p > 0.05$, Wilcoxon test, Figures 5A–5E, $n = 6$ mice) or head rotation angle compared with 500 ms before stimulation onset ($p > 0.05$, paired t test, Figures 5A and 5F–5I, $n = 6$ mice), indicating that body and head positions were kept largely immobile during stimulation.

To identify the limb pattern during stops evoked by CnF photostimulation in D_2-cre mice, we filmed mice from below in a linear corridor and used an analysis inspired by Goñi-Erro et al.,[23] who reported motor arrests when stimulating PPN $Chx10^+$ neurons (see STAR Methods). We attributed phase values to the position of each limb pair, based on the distance between left and right paws along the corridor length axis (Figures S6A–S6E). Forelimb-pair phase oscillations stopped 330 ± 77 ms after stimulation onset and resumed 1,104 ± 900 ms after stimulation offset, while hindlimb-pair phase oscillations stopped 340 ± 98 ms after stimulation onset and resumed 1,288 ± 1022 ms after stimulation offset (Video S5; Figures 5J–5L and S6A–S6E, $n = 3$ mice). At stop and resume time points, phase differences were close to zero, indicating that paws were largely immobile during motor arrest (Figures S6E and S6K). Limb pairs were almost

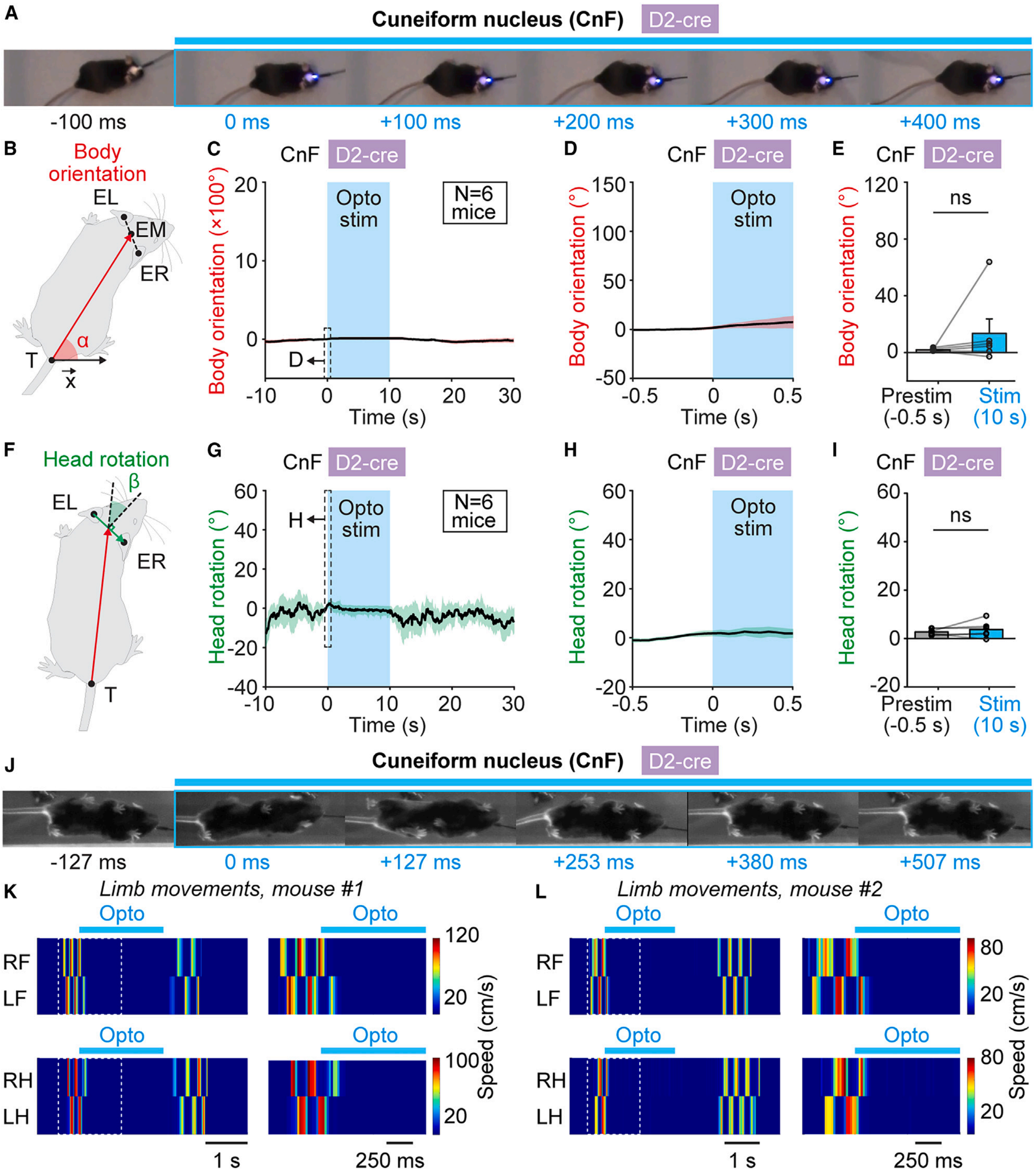

Figure 5. Body orientation, head rotation, and limb movements during arrests evoked by CnF stimulation in D_2-cre mice

(A) Example snapshots acquired from above in the open-field arena before (–100 to 0 ms) and during CnF stimulation (0–400 ms) in a D_2-cre mouse with a 470-nm laser (10-s train, 20 Hz, 10-ms pulses, 12% of laser power).

(B) Changes in body orientation were evaluated as in Usseglio et al.[22] The body orientation angle α between the x axis of the open field and the body axis vector drawn from the tail base (T) to the middle (EM) of a line drawn between left ear (EL) and right ear (ER) was calculated for each frame. To pool data from multiple trials, this angle was normalized to the average angle during the 500 ms preceding photoactivation. Angles increasing clockwise (i.e., toward the stimulated side) were considered positive.

(legend continued on next page)

systematically in stance but in various phase configurations during motor arrest (Figures S6F–S6H). To determine whether a new step cycle was initiated at movement resume, we evaluated step cycle continuity by monitoring limb-pair phase slopes, a method described by Goñi-Erro et al.[23] (Figures S6I and S6J; see STAR Methods). We observed no stereotypical pattern at movement resume, with continuity in around a third of cases and non-continuity in other cases (Figure S6L). Altogether, this indicates that CnF D_2-positive neurons strongly inhibit ongoing locomotor movements. During motor arrest, head and body orientation do not change and limb pairs can be immobilized in various stance phase configurations.

D_1-positive PPN neurons promote locomotion

We injected in the PPN of D_1-cre mice the same AAV encoding channelrhodopsin expression in a cre-dependent manner and implanted an optic fiber above the injection site (Figures S2G–S2I and S2O). Photostimulation of the PPN in D_1-cre mice increased locomotor speed in the open-field arena, increased the number of locomotor initiations, increased the time spent in locomotion, and reduced the time spent immobile (Video S6; Figures 2L–2N and S3K–S3O, $n = 4$ mice). No effect on locomotor speed was observed when replacing 470 nm light with 589 nm light (Figures S4K–S4O, $n = 4$ mice). Increasing laser power applied in the PPN increased locomotor speed (Figure 2O). We found a sigmoidal relationship between blue laser power applied to the PPN and locomotor speed in D_1-cre mice (Figure 2P, R = 0.79, $p < 0.01$, $n = 4$ mice).

Examination of body orientation and body trajectory vectors in the open field showed that D_1-cre mice were walking predominantly straight forward during PPN photostimulation, with turns occurring mainly when approaching a wall (Figures 3C, 3H, and 3M; Video S6). The average angular velocity of the trajectory was centered around zero, and there was no difference in angular velocity during stimulation compared with before or after stimulation, indicating that mice were mostly moving straight forward (Figures 3R and S5C, $n = 4$ mice).

Analysis of hindlimb kinematics during locomotion evoked by PPN stimulation in D_1-cre mice in the linear corridor showed that joint excursions during optogenetic-evoked locomotion were largely similar to those recorded during spontaneous locomotion for the hip, ankle, and metatarsophalangeal joints ($p > 0.05$, $n = 4$ mice), with the exception of the knee joint that showed slightly larger angular excursions (+19.9%, $p < 0.05$; Figures 4E and 4F, $n = 4$ mice). Altogether, this indicates that PPN D_1-positive neurons control locomotor initiation and speed, and induce forward locomotion with largely normal hindlimb movements, a role quite similar to that of CnF D_1-positive neurons.

D_2-positive PPN neurons induce ipsilateral turns

In D_2-cre mice, we injected in the PPN the same AAV encoding channelrhodopsin expression in a cre-dependent manner and implanted an optic fiber above the injection site (Figures S2J–S2L and S2P, $n = 8$ mice). Strikingly, PPN photostimulation in D_2-cre mice neurons with 470 nm light induced ipsilateral turning during the 10 s of stimulation in the open-field arena (Video S7; Figures 2Q and 2R). These turns could also be induced in the linear corridor (Video S8). This turning behavior translated into a slight but significant increase in body center speed in the open-field arena (Figures 2S, S3P, and S3Q, $n = 8$ mice), but no significant increase in the number of locomotor initiations or in the time spent in locomotion (Figures S3R–S3T, $n = 8$ mice). Gradually increasing the laser power applied to the PPN did not gradually increase locomotor speed in D_2-cre mice ($p > 0.05$, Figures 2T and 2U, $n = 8$ mice). No significant increase in body center speed was seen with 589 nm light photostimulation (Figures S4P–S4T, $n = 8$ mice).

Examination of body orientation and body trajectory vectors in the open field showed that mice were turning ipsilaterally to the stimulation side (Figures 3D, 3I, and 3N). The average angular velocity of the trajectory was significantly increased during PPN photostimulation in D_2-cre mice (Figures 3S; S5D, $n = 8$ mice), indicating that mice were turning ipsilaterally to stimulation. This effect was not seen with the three other dopamine-sensitive cell populations, as shown by the lack of significant modulation of angular velocity by optogenetic stimulation (Figures S5A–S5C). In D_2-cre mice, increasing laser power applied to PPN increased angular velocity according to a sigmoidal relationship (R = 0.89, $p < 0.0001$, Figure S5E, $n = 8$ mice). This indicates that the level of activation of D_2-positive neurons in the PPN neurons controls the angular velocity of ipsilateral turns.

(C) Changes in body orientation angle (mean ± SEM) during photoactivation of the CnF with a 470-nm laser (10-s train, 20 Hz, 10-ms pulses, 8%–12% of laser power, 10 trials per mouse, $n = 6$ mice). The dashed rectangle delineates a 500-ms time window that was extracted and illustrated in (D).

(D) Changes in body orientation angle (mean ± SEM) during the first 500 ms of photoactivation.

(E) Comparison of peak body orientation angle (mean ± SEM) during the 500 ms before photostimulation onset and 10 s after stimulation onset ($n = 6$ mice). ns, not significant, Wilcoxon test.

(F) Changes in head rotation were evaluated as in Usseglio et al.[22] The head rotation angle β between the body axis vector (defined in B) and a line perpendicular to the vector drawn from left ear (EL) to right ear (ER) was calculated for each frame. To pool data from multiple trials, this angle was normalized to the average angle during the 500 ms preceding photoactivation.

(G) Changes in head rotation angle (mean ± SEM) during photoactivation of the CnF with a 470-nm laser (10-s train, 20 Hz, 10-ms pulses, 8%–12% of laser power, 10 trials per mouse, $n = 6$ mice). The dashed rectangle delineates a 500-ms time window that was extracted and illustrated in (H).

(H) Changes in head rotation angle during the first 500 ms of photoactivation.

(I) Comparison of peak head rotation angle (mean ± SEM) during the 500 ms before photostimulation onset and 10 s after stimulation onset ($n = 6$ mice). ns, not significant, paired t test.

(J) Example snapshots acquired from below in the linear corridor before (–127 to 0 ms) and during CnF stimulation (0–507 ms) in a D_2-cre mouse with a 470-nm laser (10-s train, 20 Hz, 10-ms pulses, 10% of laser power).

(K and L) Color plots illustrating forelimb (LF, left forelimb; RF, right forelimb) and hindlimb (LH, left hindlimb; RH, right hindlimb) paw speed (warmer colors indicate higher speed) in two example mice before, during, and after CnF stimulation in D_2-cre mice with a 470-nm laser (10-s train, 20 Hz, 10-ms pulses; K, 10% and L, 12% of laser power). On the left in (K) and (L), the dashed rectangles correspond to the magnifications on the right. See also Figure S6.

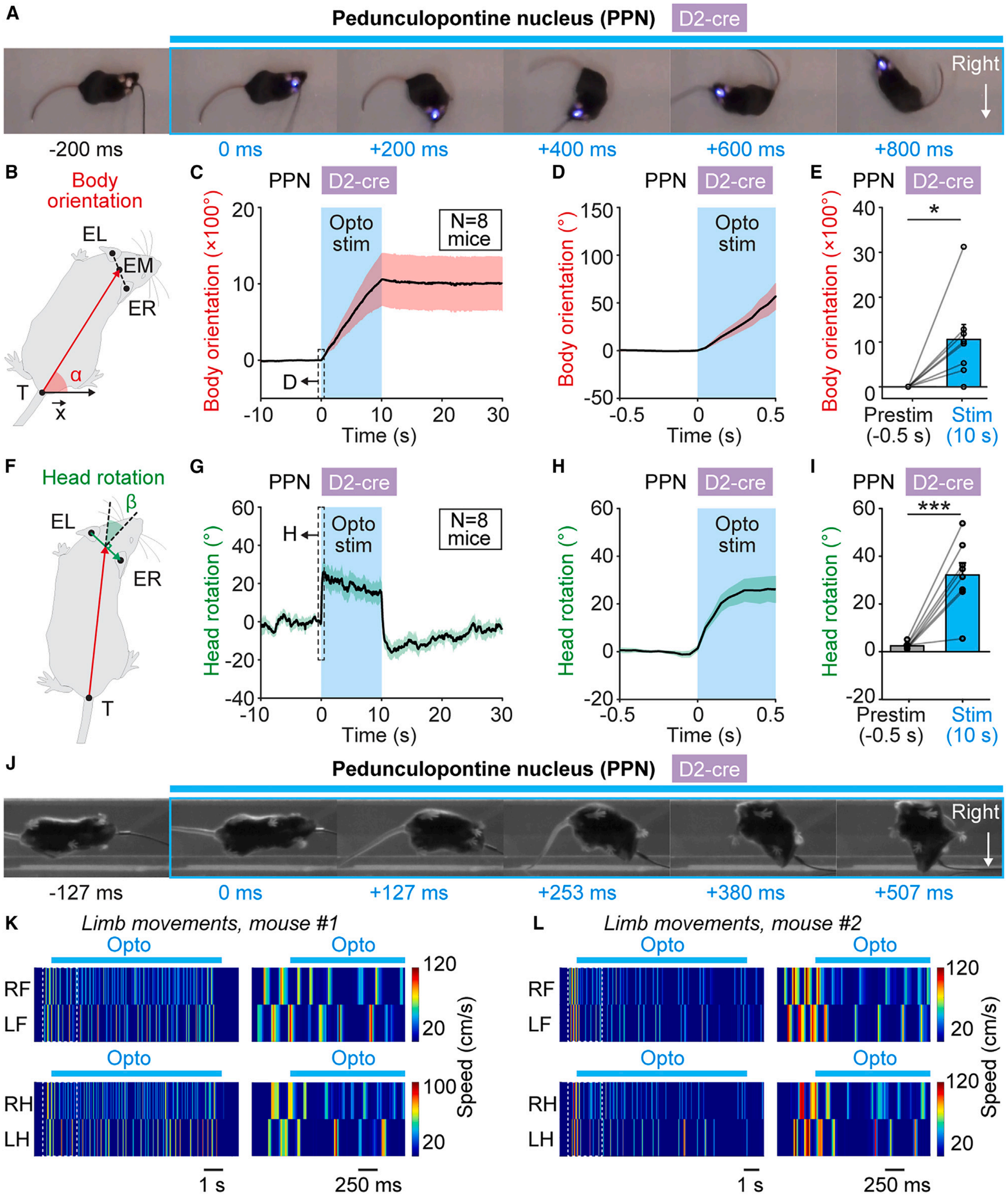

Figure 6. Body orientation, head rotation, and limb movements during arrests evoked by PPN stimulation in D_2-cre mice

(A) Example snapshots acquired from above in the open-field arena before (–200 to 0 ms) and during PPN stimulation (0–800 ms) in a D_2-cre mouse with a 470-nm laser (10-s train, 20 Hz, 10-ms pulses, 12% of laser power).

(B) Changes in body orientation were evaluated as in Usseglio et al.[22] The body orientation angle α between the x axis of the open field and the body axis vector drawn from the tail base (T) to the middle (EM) of a line drawn between left ear (EL) and right ear (ER) was calculated for each frame. To pool data from multiple

(legend continued on next page)

We also examined body orientation and head rotation angles during such turns using an analysis inspired by Usseglio et al.[22] who reported turning behavior when stimulating a subpopulation of Chx10$^+$ neurons in the gigantocellularis nucleus (see STAR Methods). PPN photostimulation in D_2-cre mice for 10 s shifted body orientation toward the stimulated side ($p < 0.05$, paired t test, Figures 6A–6E, $n = 8$ mice) and increased head rotation toward the stimulated side compared with 500 ms before stimulation onset ($p < 0.001$, paired t test, Figures 6A and 6F–6I, $n = 8$ mice). Forelimbs and hindlimbs were active during such turns (Figures 6J–6L; Video S8). Altogether, this indicates that during turns evoked by PPN D_2-positive neuron activation, head rotation and body orientation are directed toward the stimulated side, and limbs are active.

D_1 and D_2 receptor mRNA expression in MLR neurons

We examined the expression of D_1 and D_2 receptors in glutamatergic (positive for vesicular glutamate transporter 2 mRNA, Vglut2$^+$), GABAergic (positive for vesicular GABA transporter mRNA, VGAT$^+$), and cholinergic (positive for choline acetyltransferase mRNA, ChAT$^+$) MLR neurons using RNAscope (Figure 7A). In the CnF, we found D_1 receptor mRNA in 32.5% of Vglut2$^+$ cells (Figure 7B, top, $n = 3$ mice) and in 13.6% of VGAT$^+$ cells (Figure 7C, top, $n = 3$ mice). In the CnF, we found D_2 receptor mRNA in 40.7% of Vglut2$^+$ cells (Figure 7B, bottom, $n = 4$ mice) and in 32.5% of VGAT$^+$ cells (Figure 7C, bottom, $n = 4$ mice). Among neurons positive for D_1 receptor mRNA in the CnF, 75.4% were Vglut2$^+$ (Figure S7A, $n = 3$ mice) and 32.9% were VGAT$^+$ (Figure S7B, $n = 3$ mice). Among neurons positive for D_2 receptor mRNA in the CnF, 62.4% were Vglut2$^+$ (Figure S7F, $n = 4$ mice) and 36.4% were VGAT$^+$ (Figure S7G, $n = 4$ mice).

In the PPN, we found D_1 receptor mRNA in 33.3% of Vglut2$^+$ cells (Figure 7E, top, $n = 3$ mice), 17.1% of VGAT$^+$ cells (Figure 7F, top, $n = 3$ mice) and 2.6% of ChAT$^+$ cells (Figure 7D, top, $n = 4$ mice). In the PPN, we found D_2 receptor mRNA in 50.6% of Vglut2$^+$ cells (Figure 7E, bottom, $n = 3$ mice), 30.8% of VGAT$^+$ cells (Figure 7F, bottom, $n = 4$ mice), and 27.2% of ChAT$^+$ cells (Figure 7D, bottom, $n = 4$ mice). Among neurons positive for D_1 receptor mRNA in the PPN, 77.0% were Vglut2$^+$ (Figure S7C, $n = 3$ mice), 18.9% were VGAT$^+$ (Figure S7D, $n = 3$ mice) and 2.9% were ChAT$^+$ (Figure S7E, $n = 4$ mice). Among neurons positive for D_2 receptor mRNA in the PPN, 90.3% were Vglut2$^+$ (Figure S7H, $n = 3$ mice), 37.8% were VGAT$^+$ (Figure S7I, $n = 4$ mice) and 16.4% were ChAT$^+$ (Figure S7J, $n = 4$ mice). Altogether, this indicates that MLR neurons positive for D_1 or D_2 receptors comprise a combination of cells containing different neurotransmitters, with glutamatergic and GABAergic neurons being the most represented cell types.

DISCUSSION

In this study, we have identified genetically defined dopamine-sensitive cells in the mammalian MLR that control distinct aspects of movement (Figure S8). D_1-expressing neurons in the CnF and PPN control locomotion initiation and locomotor speed. D_2-expressing neurons in the CnF stop locomotion. D_2-expressing neurons in the PPN control ipsilateral turns.

Behavioral roles of dopamine-sensitive MLR neurons

Our work indicates that behaviorally relevant MLR cell types can be identified on the basis of D_1 or D_2 receptor expression. Similarly, in the basal ganglia, the expression of D_1 or D_2 receptors segregates striatal neurons of the direct and indirect pathways that respectively promote or reduce movement.[9] In the superior colliculus, a brainstem region controlling orienting movements toward salient stimuli, D_1 and D_2 receptor expression segregates distinct functional subcircuits from lamprey to mammals.[24–26] This phenomenon thus appears to be a common principle that defines the functional architecture of dopamine-sensitive motor circuits.

Our work shows that D_1-positive PPN neurons initiate locomotion, as has been demonstrated for some glutamatergic neurons in the caudal PPN.[18,27] It is also well established that other PPN glutamatergic neurons stop locomotion.[19,28,29] It is unlikely that these stop neurons express D_1 receptors, but that remains to be tested.

Some aspects of the motor arrests evoked by CnF D_2^+ neurons resemble the "pause-and-play" phenomenon evoked by

trials, this angle was normalized to the average angle during the 500 ms preceding photoactivation. Angles increasing clockwise (i.e., toward the stimulated side) were considered positive.

(C) Changes in body orientation angle (mean ± SEM) during photoactivation of the PPN with a 470-nm laser (10-s train, 20 Hz, 10-ms pulses, 10%–31% of laser power, 10 trials per mouse, $n = 8$ mice). The dashed rectangle delineates a 500-ms time window that was extracted and illustrated in (D).

(D) Changes in body orientation angle during the first 500 ms of photoactivation.

(E) Comparison of peak body orientation angle (mean ± SEM) during the 500 ms before photostimulation onset and 10 s after stimulation onset ($n = 8$ mice). *$p < 0.05$, paired t test.

(F) Changes in head rotation were evaluated as in Usseglio et al.[22] The head rotation angle β between the body axis vector (defined in B) and a line perpendicular to the vector drawn from left ear (EL) to right ear (ER) was calculated for each frame. To pool data from multiple trials, this angle was normalized to the average angle during the 500 ms preceding photoactivation.

(G) Changes in head rotation angle (mean ± SEM) during photoactivation of the PPN with a 470-nm laser (10-s train, 20 Hz, 10-ms pulses, 10%–31% of laser power, 10 trials per mouse, $n = 8$ mice). The dashed rectangle delineates a 500-ms time window that was extracted and illustrated in (H).

(H) Changes in head rotation angle during the first 500 ms of photoactivation.

(I) Comparison of peak head rotation angle during the 500 ms (mean ± SEM) before photostimulation onset and 10 s after stimulation onset ($n = 8$ mice). ***$p < 0.001$, paired t test.

(J) Example snapshots acquired from below in the linear corridor before (–127 to 0 ms) and during PPN stimulation (0–507 ms) in a D_2-cre mouse with a 470-nm laser (10-s train, 20 Hz, 10-ms pulses, 31% of laser power).

(K and L) Color plots illustrating forelimb (LF, left forelimb; RF, right forelimb) and hindlimb (LH, left hindlimb; RH, right hindlimb) paw speed (warmer colors indicate higher speed) in two example mice before, during and after PPN stimulation in D_2-cre mice with a 470-nm laser (10-s train, 20 Hz, 10-ms pulses, K, 12% and L, 31% of laser power). On the left in (K) and (L), the dashed rectangles correspond to the magnifications on the right.

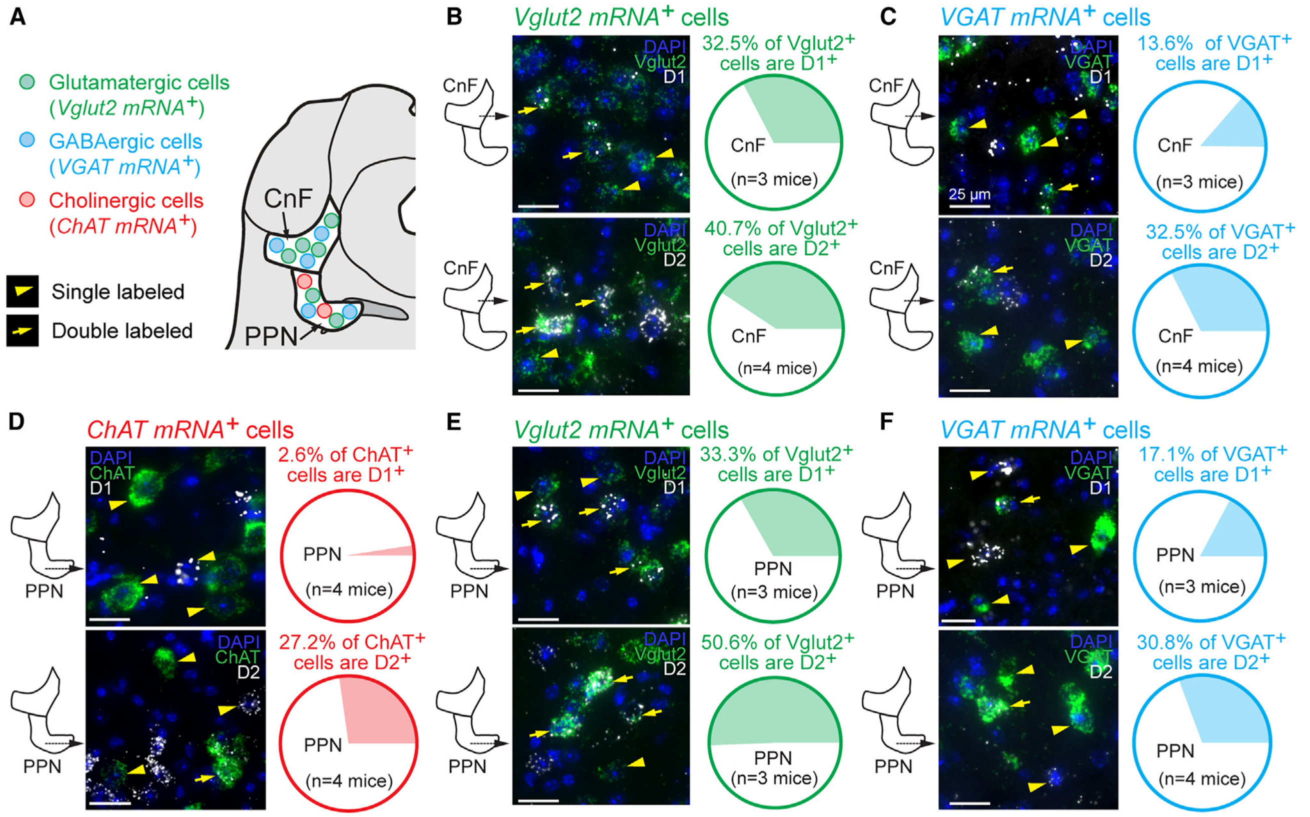

Figure 7. Neurons in the CnF or PPN expressing D_1 or D_2 receptors

(A) Scheme illustrating the neurotransmitter-based cell types of interest. Yellow arrowheads point at neurons expressing a single messenger RNA (mRNA). Yellow arrows point at cells co-expressing two mRNAs.

(B and E) Double labeling against the *vesicular glutamatergic transporter 2* (*Vglut2*) *mRNA* and *D_1 receptor mRNA* in the CnF (top B) or in the PPN (top E), or against *Vglut2 mRNA* and *D_2 receptor mRNA* in the CnF (bottom B) or in the PPN (bottom E). On the right, proportions of Vglut2$^+$ cells expressing *D_1 receptor mRNA* in the CnF (B, 18/55 Vglut2$^+$ cells in mouse 1, 58/171 in mouse 2, 36/138 in mouse 3) or PPN (E, 31/126 Vglut2$^+$ cells in mouse 1, 54/135 in mouse 2, 58/160 in mouse 3), or expressing *D_2 receptor mRNA* in the CnF (B, 20/58 Vglut2$^+$ cells in mouse 1, 47/140 in mouse 2, 23/48 in mouse 3, 15/32 in mouse 4) or PPN (E, 58/120 Vglut2$^+$ cells in mouse 1, 25/45 in mouse 2, 12/25 in mouse 3).

(C and F) Double labeling against the *vesicular GABAergic transporter (VGAT) mRNA* and *D_1 receptor mRNA* in the CnF (top C) or in the PPN (top F), or against *VGAT mRNA* and *D_2 receptor mRNA* in the CnF (bottom C) or in the PPN (bottom F). On the right, proportions of VGAT$^+$ cells expressing *D_1 receptor mRNA* in the CnF (C, 13/78 VGAT$^+$ cells in mouse 1, 14/120 in mouse 2, 10/70 in mouse 3) or PPN (F, 3/43 VGAT$^+$ cells in mouse 1, 12/57 in mouse 2, 9/38 in mouse 3) or *D_2 receptor mRNA* in the CnF (C, 15/62 VGAT$^+$ cells in mouse 1, 35/90 in mouse 2, 23/74 in mouse 3, 33/104 in mouse 4) or PPN (F, 21/84 VGAT$^+$ cells in mouse 1, 28/65 in mouse 2, 15/60 in mouse 3, 18/54 in mouse 4).

(D) Double labeling against the *choline acetyltransferase (ChAT) mRNA* and *D_1 receptor mRNA* (top), or against *ChAT mRNA* and *D_2 receptor mRNA* (bottom) in the PPN. On the right, proportions of ChAT$^+$ neurons expressing *D_1 receptor mRNA* (top, 0/19 ChAT$^+$ cells in mouse 1, 1/17 in mouse 2, 1/70 in mouse 3, 2/59 in mouse 4) or *D_2 receptor mRNA* (bottom, 3/16 ChAT$^+$ cells in mouse 1, 8/53 in mouse 2, 17/58 in mouse 3, 18/43 in mouse 4). (B–F) Quantifications made from 1 to 3 slices per mouse. Scale bars, 25 μm. See also Figure S7.

PPN Chx10 neuron stimulation.[23] We found that step cycle continuity was maintained in around a third of the trials, and that limbs could be immobilized in a variety of stance phase configurations. However, we rarely observed a limb immobilized in swing phase, a common observation when stimulating PPN Chx10 neurons.[23] The limb patterns during CnF D_2^+-evoked motor arrests appear to be more diverse than the stereotypical, symmetrical left-right limb pattern evoked either by Chx10 neurons in the periaqueductal gray[23] or by Chx10 gigantocellularis nucleus neurons projecting to forelimb spinal segments.[22,30] The absence of change in body orientation and head rotation during CnF D_2^+-evoked motor arrests resembles the effect of gigantocellularis nucleus Chx10 neurons projecting to hindlimb spinal segments.[22] The latency to arrest for CnF D_2^+-evoked motor arrests (330 ± 77 ms for hindlimbs) is around two times higher than those reported for Chx10 neurons in the PPN, periaqueductal gray, or gigantocellularis nucleus, suggesting that some of these regions could be targeted by the projections of CnF D_2^+ neurons.

The turns evoked by PPN D_2^+ neurons were associated with changes in body orientation (49° ± 11° at 500 ms of stimulation) and head rotation (28° ± 5° at 500 ms of stimulation) toward the stimulated side. These values are in the ranges reported when stimulating gigantocellularis nucleus Chx10 neurons projecting to forelimb spinal segments.[22] This suggests that gigantocellularis nucleus Chx10 neurons may be targeted by PPN D_2^+ neurons.

Circuit architecture

Our data indicate that dopaminergic receptor expression likely defines functional microcircuits within neurotransmitter-based cell types of the MLR. Our work shows that D_1-positive and D_2-positive neurons in the MLR contain a combination of glutamatergic, GABAergic, and cholinergic neurons. These observations are consistent with recent single-cell transcriptomic data showing that D_1 mRNA and D_2 mRNA are present in CnF and PPN neurons expressing Vglut2, VGAT, or ChAT mRNA.[31,32] These neurotransmitter-based cell populations likely act in synergy to produce coherent behavioral output. In the spinal cord, a microcircuit composed of V2 lineage-derived excitatory (V2a) and inhibitory (V2b) neurons together coordinate ipsilateral body movements during locomotion.[33] In the MLR, the control of distinct behaviors could rely on a modular architecture, with each module comprising a group of glutamatergic neurons that activates a command network downstream in the reticular formation, and a group of GABAergic neurons that inhibits other modules.

The role of the cholinergic neurons expressing dopaminergic receptors remains to be identified. In lampreys, cholinergic MLR neurons provide additional excitation to reticulospinal neurons directly through nicotinic receptors and indirectly through muscarinoceptive neurons and thereby increase locomotor speed.[34] In mammals, their stimulation can moderately increase,[5,18] decrease,[19] or have no effect on speed[35] (for review, see Ryczko[3]). Such diversity may relate to the complex projections of PPN cholinergic neurons that include striatum,[36–39] dopaminergic neurons,[40,41] or thalamus,[42] among other regions (for review, see Mena-Segovia and Bolam[43]). Whether dopamine-sensitive PPN cholinergic cells play a role in sleep or arousal[44,45] or cognitive flexibility such as reversal learning[35] remains to be determined.

Future studies should examine the projection patterns of dopamine-sensitive MLR cell types to determine how they produce behavioral output. CnF and PPN D_1-positive neurons, which promote locomotion, likely contain glutamatergic neurons projecting to the lateral paragigantocellular nucleus, which are known to induce locomotion.[46] D_2-positive CnF neurons, which stop locomotion, could do so by sending glutamatergic input to neurons known to stop locomotion, such as PPN Chx10 neurons or periaqueductal gray Chx10 neurons,[23] PPN glutamatergic neurons projecting to basal ganglia output stations,[47] Chx10 neurons of the gigantocellularis nucleus,[22,30] or glycinergic neurons in the lateral paragigantocellular nucleus.[46] D_2-positive CnF neurons could also contain GABAergic neurons that stop locomotion,[18] and that likely act through inhibition of locomotion-inducing glutamatergic neurons.[5] D_2-positive PPN neurons that produce ipsilateral turns could do so by sending glutamatergic input to ipsilateral gigantocellularis nucleus Chx10 neurons,[48] or to the striatum neurons that produce ipsilateral head turns.[49] Within each class of dopamine-sensitive neurons, GABAergic neurons may contribute to switching between motor commands through competitive inhibition as has been proposed in other brain regions.[50–52]

A motor substrate for dopamine

Dopamine release has been recorded in the MLR using voltammetry from lamprey to mammals.[10,11] Our study uncovers a substrate in the MLR by which dopamine can control movement in mammals. Importantly, activation of dopamine-sensitive neurons is not equivalent to the effects of dopamine on those neurons. In lamprey, dopamine release in the MLR increases the duration of the locomotor bout, the frequency of locomotor movements, and the number of locomotor cycles through the activation of D_1 receptors[10,12] (for review, see Ryczko and Dubuc [15]). In the mammalian MLR, dopamine likely promotes locomotion by increasing the excitability of D_1-expressing pro-locomotor neurons in the CnF and PPN, and by decreasing the excitability in D_2-expressing neurons that stop locomotion. In the PPN, dopamine also likely decreases turning by decreasing the excitability of D_2-expressing neurons that control turning. Dopamine release in the MLR is potentiated *in vivo* by systemic application of amphetamine in rats.[11] The cell types reported here are well positioned to contribute to the hyperlocomotor effects of psychostimulants.

Pathology

Dopaminergic innervation of the MLR is present in lamprey, salamander, rat and mouse,[10–14] monkey,[53] and human.[11] In monkeys treated with the neurotoxin MPTP, used for decades to model Parkinson's disease, dopaminergic fibers disappear in the MLR.[53] The loss of dopaminergic input to the MLR should result in a loss of amplification of locomotor commands, likely contributing to locomotor deficits. This would be in accordance with studies in lamprey showing that blockade of D_1 receptors in the MLR decreases locomotor movements evoked by stimulation of the meso-diencephalic dopaminergic region.[10,12] Patients with Parkinson's disease display dysfunctions in locomotor initiation, stops and turns[54] (for review, see Ryczko and Dubuc[55]), all of which are controlled by the MLR cell types we report here. Our work suggests that dopaminergic drugs such as levodopa and dopaminergic agonists, which are both used to improve locomotor function in Parkinson's disease, directly influence the excitability of the MLR neurons uncovered here.

Limitations of the study

Concerning photostimulation, we cannot rule out partial activation of the PPN when targeting the CnF or vice versa because of their proximity, or potential recruitment of neighboring regions involved in motor control, such as the periaqueductal gray,[23] mesencephalic reticular formation,[47] pontine nucleus oralis,[56] or dorsal raphe.[57] To evaluate the extent of possible off-target activation, we estimated the light cone angle (18.6°) emanating from the optic fiber using calculations taking into account fiber numerical aperture (0.22) and refractive index of brain tissue (1.36)[58] (Figures S2C, S2F, S2I, and S2L; see STAR Methods). For each animal included in the present study, we estimated the loss of light irradiance as a function of distance from fiber tip based on measurements made in brain tissue[59] (Figures S2M–S2P). It is estimated that most light irradiance (~98%) is lost 1 mm away from the fiber[19,21,27,59] (see STAR Methods). We also cannot rule out the possibility that light delivered in the MLR recruited non-dopamine-sensitive MLR neurons that receive afferents from dopamine-sensitive neurons in the structures mentioned above, or antidromically activated cell bodies from these structures, which in turn elicited motor effects by recruiting parallel downstream motor circuits.

Concerning our quantifications of MLR cells expressing D_1^+ receptor, D_2^+ receptor, Vglut2, VGAT, and ChAT mRNAs, we mixed brain sections from different rostrocaudal levels and did not take into account cell type heterogeneity across the rostrocaudal axis. Neuronal markers are heterogeneously distributed rostrocaudally, especially in the PPN.[23,31,32,60–62] We also did not take into account the possibility that some neurons may use multiple neurotransmitters, and thereby co-express, e.g., Vglut2 and ChAT, or VGAT and ChAT.[63,64]

STAR★METHODS

Detailed methods are provided in the online version of this paper and include the following:

- KEY RESOURCES TABLE
- RESOURCE AVAILABILITY
  - Lead contact
  - Materials availability
  - Data and code availability
- EXPERIMENTAL MODEL AND STUDY PARTICIPANT DETAILS
  - Ethics statement
  - Animals
- METHOD DETAILS
  - Virus injection and optic fiber implantation
  - *In vivo* optogenetic stimulation
  - Open-field locomotion
  - Linear corridor and limb kinematics
  - DeepLabCut networks
  - Treadmill
  - Histology
  - Immunofluorescence
  - RNAscope
  - Microscopy
  - Cell counting
- QUANTIFICATION AND STATISTICAL ANALYSIS

SUPPLEMENTAL INFORMATION

Supplemental information can be found online at https://doi.org/10.1016/j.celrep.2024.114187.

ACKNOWLEDGMENTS

This work was supported by the Canadian Institutes of Health Research (407083 to D.R.), the Natural Sciences and Engineering Research Council of Canada (RGPIN-2017-05522 and RTI-2019-00628 to D.R.), the Fonds de la Recherche du Québec - Santé (FRQS Junior 1 awards 34920 and 36772, and Junior 2 award 297238 to D.R.; Doctoral training scholarship 335559 to C.I.v.d.Z.; Postdoctoral training fellowship 332188 to J.S.); the Canada Foundation for Innovation (39344 to D.R.), the Centre de Recherche du Centre Hospitalier Universitaire de Sherbrooke (start-up funding and PAFI grant to D.R.), the Faculté de médecine et des sciences de la santé (start-up funding to D.R.), the Centre d'excellence en Neurosciences de l'Université de Sherbooke (to D.R.), and the fonds Jean-Luc Mongrain de la Fondation du CHUS (to D.R.). This study has received funding from the European Research Council under the European Union's Horizon 2020 research and innovation programme (grant agreement no. 951477 to D.R.). We thank Prof. Gendron for providing access to the stimulator and treadmill, Prof. Denault for providing access to a −80°C freezer, and Prof. Valjent for his input on the choice of the transgenic mice used in this study.

AUTHOR CONTRIBUTIONS

Conceptualization, A.J.T., C.I.v.d.Z., L.D., and D.R.; data curation, A.J.T., C.I.v.d.Z., L.D., J.B., J.D.-Y., K.M.-O., J.S.S., J.S., and D.R.; formal analysis, A.J.T., C.I.v.d.Z., L.D., K.M.-O., J.S.S., and D.R.; investigation, A.J.T., C.I.v.d.Z., L.D., J.B., J.D.-Y., and J.S.; methodology, A.J.T., C.I.v.d.Z., J.B., J.D.-Y., J.S., and D.R.; visualization, A.J.T., C.I.v.d.Z., L.D., and D.R.; validation, A.J.T., C.I.v.d.Z., L.D., and D.R.; writing – original draft, A.J.T., C.I.v.d.Z., and D.R.; writing – review & editing, A.J.T., C.I.v.d.Z., L.D., J.B., J.D.-Y., J.S., K.M.-O., J.S.S., P.S., and D.R.; software, C.I.v.d.Z.; resources, P.S. and D.R.; funding acquisition, project administration, and supervision, D.R. All authors contributed to the article and approved the submitted version.

DECLARATION OF INTERESTS

The authors declare no competing interests.

Received: October 20, 2023
Revised: March 31, 2024
Accepted: April 17, 2024
Published: May 8, 2024

REFERENCES

1. Arber, S., and Costa, R.M. (2022). Networking brainstem and basal ganglia circuits for movement. Nat. Rev. Neurosci. *23*, 342–360. https://doi.org/10.1038/s41583-022-00581-w.
2. Leiras, R., Cregg, J.M., and Kiehn, O. (2022). Brainstem Circuits for Locomotion. Annu. Rev. Neurosci. *45*, 63–85. https://doi.org/10.1146/annurev-neuro-082321-025137.
3. Ryczko, D. (2022). The Mesencephalic Locomotor Region: Multiple Cell Types, Multiple Behavioral Roles, and Multiple Implications for Disease. Neuroscientist, 10738584221139136. https://doi.org/10.1177/10738584221139136.
4. Dubuc, R., Cabelguen, J.-M., and Ryczko, D. (2023). Locomotor pattern generation and descending control: a historical perspective. J. Neurophysiol. *130*, 401–416. https://doi.org/10.1152/jn.00204.2023.
5. Roseberry, T.K., Lee, A.M., Lalive, A.L., Wilbrecht, L., Bonci, A., and Kreitzer, A.C. (2016). Cell-Type-Specific Control of Brainstem Locomotor Circuits by Basal Ganglia. Cell *164*, 526–537. https://doi.org/10.1016/j.cell.2015.12.037.
6. Howe, M.W., and Dombeck, D.A. (2016). Rapid signalling in distinct dopaminergic axons during locomotion and reward. Nature *535*, 505–510. https://doi.org/10.1038/nature18942.
7. da Silva, J.A., Tecuapetla, F., Paixão, V., and Costa, R.M. (2018). Dopamine neuron activity before action initiation gates and invigorates future movements. Nature *554*, 244–248. https://doi.org/10.1038/nature25457.
8. Markowitz, J.E., Gillis, W.F., Jay, M., Wood, J., Harris, R.W., Cieszkowski, R., Scott, R., Brann, D., Koveal, D., Kula, T., et al. (2023). Spontaneous behaviour is structured by reinforcement without explicit reward. Nature *614*, 108–117. https://doi.org/10.1038/s41586-022-05611-2.
9. Kravitz, A.V., Freeze, B.S., Parker, P.R.L., Kay, K., Thwin, M.T., Deisseroth, K., and Kreitzer, A.C. (2010). Regulation of parkinsonian motor behaviours by optogenetic control of basal ganglia circuitry. Nature *466*, 622–626. https://doi.org/10.1038/nature09159.
10. Ryczko, D., Grätsch, S., Auclair, F., Dubé, C., Bergeron, S., Alpert, M.H., Cone, J.J., Roitman, M.F., Alford, S., and Dubuc, R. (2013). Forebrain dopamine neurons project down to a brainstem region controlling locomotion. Proc. Natl. Acad. Sci. USA *110*, E3235–E3242. https://doi.org/10.1073/pnas.1301125110.
11. Ryczko, D., Cone, J.J., Alpert, M.H., Goetz, L., Auclair, F., Dubé, C., Parent, M., Roitman, M.F., Alford, S., and Dubuc, R. (2016). A descending dopamine pathway conserved from basal vertebrates to mammals. Proc. Natl. Acad. Sci. USA *113*, E2440–E2449. https://doi.org/10.1073/pnas.1600684113.
12. Ryczko, D., Grätsch, S., Schläger, L., Keuyalian, A., Boukhatem, Z., Garcia, C., Auclair, F., Büschges, A., and Dubuc, R. (2017). Nigral Glutamatergic

CellPress
OPEN ACCESS

Neurons Control the Speed of Locomotion. J. Neurosci. *37*, 9759–9770. https://doi.org/10.1523/JNEUROSCI.1810-17.2017.

13. Pérez-Fernández, J., Stephenson-Jones, M., Suryanarayana, S.M., Robertson, B., and Grillner, S. (2014). Evolutionarily conserved organization of the dopaminergic system in lamprey: SNc/VTA afferent and efferent connectivity and D2 receptor expression. J. Comp. Neurol. *522*, 3775–3794. https://doi.org/10.1002/cne.23639.
14. Sharma, S., Kim, L.H., Mayr, K.A., Elliott, D.A., and Whelan, P.J. (2018). Parallel descending dopaminergic connectivity of A13 cells to the brainstem locomotor centers. Sci. Rep. *8*, 7972. https://doi.org/10.1038/s41598-018-25908-5.
15. Ryczko, D., and Dubuc, R. (2023). Dopamine control of downstream motor centers. Curr. Opin. Neurobiol. *83*, 102785. https://doi.org/10.1016/j.conb.2023.102785.
16. Gong, S., Zheng, C., Doughty, M.L., Losos, K., Didkovsky, N., Schambra, U.B., Nowak, N.J., Joyner, A., Leblanc, G., Hatten, M.E., and Heintz, N. (2003). A gene expression atlas of the central nervous system based on bacterial artificial chromosomes. Nature *425*, 917–925. https://doi.org/10.1038/nature02033.
17. Gong, S., Doughty, M., Harbaugh, C.R., Cummins, A., Hatten, M.E., Heintz, N., and Gerfen, C.R. (2007). Targeting Cre recombinase to specific neuron populations with bacterial artificial chromosome constructs. J. Neurosci. *27*, 9817–9823. https://doi.org/10.1523/JNEUROSCI.2707-07.2007.
18. Caggiano, V., Leiras, R., Goñi-Erro, H., Masini, D., Bellardita, C., Bouvier, J., Caldeira, V., Fisone, G., and Kiehn, O. (2018). Midbrain circuits that set locomotor speed and gait selection. Nature *553*, 455–460. https://doi.org/10.1038/nature25448.
19. Josset, N., Roussel, M., Lemieux, M., Lafrance-Zoubga, D., Rastqar, A., and Bretzner, F. (2018). Distinct Contributions of Mesencephalic Locomotor Region Nuclei to Locomotor Control in the Freely Behaving Mouse. Curr. Biol. *28*, 884–901.e3. https://doi.org/10.1016/j.cub.2018.02.007.
20. van der Zouwen, C.I., Boutin, J., Fougère, M., Flaive, A., Vivancos, M., Santuz, A., Akay, T., Sarret, P., and Ryczko, D. (2021). Freely Behaving Mice Can Brake and Turn During Optogenetic Stimulation of the Mesencephalic Locomotor Region. Front. Neural Circ. *15*, 639900. https://doi.org/10.3389/fncir.2021.639900.
21. Fougère, M., van der Zouwen, C.I., Boutin, J., Neszvecsko, K., Sarret, P., and Ryczko, D. (2021). Optogenetic stimulation of glutamatergic neurons in the cuneiform nucleus controls locomotion in a mouse model of Parkinson's disease. Proc. Natl. Acad. Sci. USA *118*, e2110934118. https://doi.org/10.1073/pnas.2110934118.
22. Usseglio, G., Gatier, E., Heuzé, A., Hérent, C., and Bouvier, J. (2020). Control of Orienting Movements and Locomotion by Projection-Defined Subsets of Brainstem V2a Neurons. Curr. Biol. *30*, 4665–4681.e6. https://doi.org/10.1016/j.cub.2020.09.014.
23. Goñi-Erro, H., Selvan, R., Leiras, R., and Kiehn, O. (2023). Pedunculopontine Chx10+ neurons control global motor arrest in mice. Nat. Neurosci. *26*, 1516–1528. https://doi.org/10.1038/s41593-023-01396-3.
24. Pérez-Fernández, J., Kardamakis, A.A., Suzuki, D.G., Robertson, B., and Grillner, S. (2017). Direct Dopaminergic Projections from the SNc Modulate Visuomotor Transformation in the Lamprey Tectum. Neuron *96*, 910–924.e5. https://doi.org/10.1016/j.neuron.2017.09.051.
25. von Twickel, A., Kowatschew, D., Saltürk, M., Schauer, M., Robertson, B., Korsching, S., Walkowiak, W., Grillner, S., and Pérez-Fernández, J. (2019). Individual Dopaminergic Neurons of Lamprey SNc/VTA Project to Both the Striatum and Optic Tectum but Restrict Co-release of Glutamate to Striatum Only. Curr. Biol. *29*, 677–685.e6. https://doi.org/10.1016/j.cub.2019.01.004.
26. Bolton, A.D., Murata, Y., Kirchner, R., Kim, S.-Y., Young, A., Dang, T., Yanagawa, Y., and Constantine-Paton, M. (2015). A Diencephalic Dopamine Source Provides Input to the Superior Colliculus, where D1 and D2 Receptors Segregate to Distinct Functional Zones. Cell Rep. *13*, 1003–1015. https://doi.org/10.1016/j.celrep.2015.09.046.
27. Masini, D., and Kiehn, O. (2022). Targeted activation of midbrain neurons restores locomotor function in mouse models of parkinsonism. Nat. Commun. *13*, 504. https://doi.org/10.1038/s41467-022-28075-4.
28. Carvalho, M.M., Tanke, N., Kropff, E., Witter, M.P., Moser, M.-B., and Moser, E.I. (2020). A Brainstem Locomotor Circuit Drives the Activity of Speed Cells in the Medial Entorhinal Cortex. Cell Rep. *32*, 108123. https://doi.org/10.1016/j.celrep.2020.108123.
29. Dautan, D., Kovács, A., Bayasgalan, T., Diaz-Acevedo, M.A., Pal, B., and Mena-Segovia, J. (2021). Modulation of motor behavior by the mesencephalic locomotor region. Cell Rep. *36*, 109594. https://doi.org/10.1016/j.celrep.2021.109594.
30. Bouvier, J., Caggiano, V., Leiras, R., Caldeira, V., Bellardita, C., Balueva, K., Fuchs, A., and Kiehn, O. (2015). Descending Command Neurons in the Brainstem that Halt Locomotion. Cell *163*, 1191–1203. https://doi.org/10.1016/j.cell.2015.10.074.
31. Yao, Z., van Velthoven, C.T.J., Kunst, M., Zhang, M., McMillen, D., Lee, C., Jung, W., Goldy, J., Abdelhak, A., Aitken, M., et al. (2023). A high-resolution transcriptomic and spatial atlas of cell types in the whole mouse brain. Nature *624*, 317–332. https://doi.org/10.1038/s41586-023-06812-z.
32. Zhang, M., Pan, X., Jung, W., Halpern, A.R., Eichhorn, S.W., Lei, Z., Cohen, L., Smith, K.A., Tasic, B., Yao, Z., et al. (2023). Molecularly defined and spatially resolved cell atlas of the whole mouse brain. Nature *624*, 343–354. https://doi.org/10.1038/s41586-023-06808-9.
33. Hayashi, M., Gullo, M., Senturk, G., Di Costanzo, S., Nagasaki, S.C., Kageyama, R., Imayoshi, I., Goulding, M., Pfaff, S.L., and Gatto, G. (2023). A spinal synergy of excitatory and inhibitory neurons coordinates ipsilateral body movements. Preprint at bioRxiv, 2023.03.21.533603. https://doi.org/10.1101/2023.03.21.533603.
34. Smetana, R., Juvin, L., Dubuc, R., and Alford, S. (2010). A parallel cholinergic brainstem pathway for enhancing locomotor drive. Nat. Neurosci. *13*, 731–738. https://doi.org/10.1038/nn.2548.
35. Ruan, Y., Li, K.-Y., Zheng, R., Yan, Y.-Q., Wang, Z.-X., Chen, Y., Liu, Y., Tian, J., Zhu, L.-Y., Lou, H.-F., et al. (2022). Cholinergic neurons in the pedunculopontine nucleus guide reversal learning by signaling the changing reward contingency. Cell Rep. *38*, 110437. https://doi.org/10.1016/j.celrep.2022.110437.
36. Brimblecombe, K.R., Threlfell, S., Dautan, D., Kosillo, P., Mena-Segovia, J., and Cragg, S.J. (2018). Targeted Activation of Cholinergic Interneurons Accounts for the Modulation of Dopamine by Striatal Nicotinic Receptors. eNeuro *5*. https://doi.org/10.1523/ENEURO.0397-17.2018.
37. Dautan, D., Huerta-Ocampo, I., Witten, I.B., Deisseroth, K., Bolam, J.P., Gerdjikov, T., and Mena-Segovia, J. (2014). A Major External Source of Cholinergic Innervation of the Striatum and Nucleus Accumbens Originates in the Brainstem. J. Neurosci. *34*, 4509–4518. https://doi.org/10.1523/JNEUROSCI.5071-13.2014.
38. Dautan, D., Huerta-Ocampo, I., Gut, N.K., Valencia, M., Kondabolu, K., Kim, Y., Gerdjikov, T.V., and Mena-Segovia, J. (2020). Cholinergic midbrain afferents modulate striatal circuits and shape encoding of action strategies. Nat. Commun. *11*, 1739. https://doi.org/10.1038/s41467-020-15514-3.
39. Klug, J.R., Engelhardt, M.D., Cadman, C.N., Li, H., Smith, J.B., Ayala, S., Williams, E.W., Hoffman, H., and Jin, X. (2018). Differential inputs to striatal cholinergic and parvalbumin interneurons imply functional distinctions. Elife *7*, e35657. https://doi.org/10.7554/eLife.35657.
40. Dautan, D., Souza, A.S., Huerta-Ocampo, I., Valencia, M., Assous, M., Witten, I.B., Deisseroth, K., Tepper, J.M., Bolam, J.P., Gerdjikov, T.V., and Mena-Segovia, J. (2016). Segregated cholinergic transmission modulates dopamine neurons integrated in distinct functional circuits. Nat. Neurosci. *19*, 1025–1033. https://doi.org/10.1038/nn.4335.
41. Xiao, C., Cho, J.R., Zhou, C., Treweek, J.B., Chan, K., McKinney, S.L., Yang, B., and Gradinaru, V. (2016). Cholinergic Mesopontine Signals Govern Locomotion and Reward through Dissociable Midbrain Pathways. Neuron *90*, 333–347. https://doi.org/10.1016/j.neuron.2016.03.028.

42. Huerta-Ocampo, I., Hacioglu-Bay, H., Dautan, D., and Mena-Segovia, J. (2020). Distribution of Midbrain Cholinergic Axons in the Thalamus. eNeuro *7*. https://doi.org/10.1523/ENEURO.0454-19.2019.

43. Mena-Segovia, J., and Bolam, J.P. (2017). Rethinking the Pedunculopontine Nucleus: From Cellular Organization to Function. Neuron *94*, 7–18. https://doi.org/10.1016/j.neuron.2017.02.027.

44. Gut, N.K., and Mena-Segovia, J. (2019). Dichotomy between motor and cognitive functions of midbrain cholinergic neurons. Neurobiol. Dis. *128*, 59–66. https://doi.org/10.1016/j.nbd.2018.09.008.

45. Ni, K.-M., Hou, X.-J., Yang, C.-H., Dong, P., Li, Y., Zhang, Y., Jiang, P., Berg, D.K., Duan, S., and Li, X.-M. (2016). Selectively driving cholinergic fibers optically in the thalamic reticular nucleus promotes sleep. Elife *5*, e10382. https://doi.org/10.7554/eLife.10382.

46. Capelli, P., Pivetta, C., Soledad Esposito, M., and Arber, S. (2017). Locomotor speed control circuits in the caudal brainstem. Nature *551*, 373–377. https://doi.org/10.1038/nature24064.

47. Ferreira-Pinto, M.J., Kanodia, H., Falasconi, A., Sigrist, M., Esposito, M.S., and Arber, S. (2021). Functional diversity for body actions in the mesencephalic locomotor region. Cell *184*, 4564–4578.e18. https://doi.org/10.1016/j.cell.2021.07.002.

48. Cregg, J.M., Leiras, R., Montalant, A., Wanken, P., Wickersham, I.R., and Kiehn, O. (2020). Brainstem neurons that command mammalian locomotor asymmetries. Nat. Neurosci. *23*, 730–740. https://doi.org/10.1038/s41593-020-0633-7.

49. Assous, M., Dautan, D., Tepper, J.M., and Mena-Segovia, J. (2019). Pedunculopontine Glutamatergic Neurons Provide a Novel Source of Feedforward Inhibition in the Striatum by Selectively Targeting Interneurons. J. Neurosci. *39*, 4727–4737. https://doi.org/10.1523/JNEUROSCI.2913-18.2019.

50. Michelet, T., Duncan, G.H., and Cisek, P. (2010). Response competition in the primary motor cortex: corticospinal excitability reflects response replacement during simple decisions. J. Neurophysiol. *104*, 119–127. https://doi.org/10.1152/jn.00819.2009.

51. Munakata, Y., Herd, S.A., Chatham, C.H., Depue, B.E., Banich, M.T., and O'Reilly, R.C. (2011). A unified framework for inhibitory control. Trends Cognit. Sci. *15*, 453–459. https://doi.org/10.1016/j.tics.2011.07.011.

52. Roach, J.P., Churchland, A.K., and Engel, T.A. (2023). Choice selective inhibition drives stability and competition in decision circuits. Nat. Commun. *14*, 147. https://doi.org/10.1038/s41467-023-35822-8.

53. Rolland, A.-S., Tandé, D., Herrero, M.-T., Luquin, M.-R., Vazquez-Claverie, M., Karachi, C., Hirsch, E.C., and François, C. (2009). Evidence for a dopaminergic innervation of the pedunculopontine nucleus in monkeys, and its drastic reduction after MPTP intoxication. J. Neurochem. *110*, 1321–1329. https://doi.org/10.1111/j.1471-4159.2009.06220.x.

54. Mellone, S., Mancini, M., King, L.A., Horak, F.B., and Chiari, L. (2016). The quality of turning in Parkinson's disease: a compensatory strategy to prevent postural instability? J. NeuroEng. Rehabil. *13*, 39. https://doi.org/10.1186/s12984-016-0147-4.

55. Ryczko, D., and Dubuc, R. (2017). Dopamine and the Brainstem Locomotor Networks: From Lamprey to Human. Front. Neurosci. *11*, 295. https://doi.org/10.3389/fnins.2017.00295.

56. Cregg, J.M., Sidhu, S.K., Leiras, R., and Kiehn, O. (2024). Basal ganglia-spinal cord pathway that commands locomotor gait asymmetries in mice. Nat. Neurosci. *27*, 716–727. https://doi.org/10.1038/s41593-024-01569-8.

57. Correia, P.A., Lottem, E., Banerjee, D., Machado, A.S., Carey, M.R., and Mainen, Z.F. (2017). Transient inhibition and long-term facilitation of locomotion by phasic optogenetic activation of serotonin neurons. Elife *6*, e20975. https://doi.org/10.7554/eLife.20975.

58. Yona, G., Meitav, N., Kahn, I., and Shoham, S. (2016). Realistic Numerical and Analytical Modeling of Light Scattering in Brain Tissue for Optogenetic Applications. eNeuro *3*. https://doi.org/10.1523/ENEURO.0059-15.2015.

59. Yizhar, O., Fenno, L.E., Davidson, T.J., Mogri, M., and Deisseroth, K. (2011). Optogenetics in neural systems. Neuron *71*, 9–34. https://doi.org/10.1016/j.neuron.2011.06.004.

60. Wang, H.-L., and Morales, M. (2009). Pedunculopontine and laterodorsal tegmental nuclei contain distinct populations of cholinergic, glutamatergic and GABAergic neurons in the rat. Eur. J. Neurosci. *29*, 340–358. https://doi.org/10.1111/j.1460-9568.2008.06576.x.

61. Mena-Segovia, J., Micklem, B.R., Nair-Roberts, R.G., Ungless, M.A., and Bolam, J.P. (2009). GABAergic neuron distribution in the pedunculopontine nucleus defines functional subterritories. J. Comp. Neurol. *515*, 397–408. https://doi.org/10.1002/cne.22065.

62. Martinez-Gonzalez, C., Wang, H.-L., Micklem, B.R., Bolam, J.P., and Mena-Segovia, J. (2012). Subpopulations of cholinergic, GABAergic and glutamatergic neurons in the pedunculopontine nucleus contain calcium-binding proteins and are heterogeneously distributed. Eur. J. Neurosci. *35*, 723–734. https://doi.org/10.1111/j.1460-9568.2012.08002.x.

63. Luquin, E., Huerta, I., Aymerich, M.S., and Mengual, E. (2018). Stereological Estimates of Glutamatergic, GABAergic, and Cholinergic Neurons in the Pedunculopontine and Laterodorsal Tegmental Nuclei in the Rat. Front. Neuroanat. *12*, 34. https://doi.org/10.3389/fnana.2018.00034.

64. Steinkellner, T., Yoo, J.H., and Hnasko, T.S. (2019). Differential Expression of VGLUT2 in Mouse Mesopontine Cholinergic Neurons. eNeuro *6*. https://doi.org/10.1523/ENEURO.0161-19.2019.

65. Fougère, M., van der Zouwen, C.I., Boutin, J., and Ryczko, D. (2021). Heterogeneous expression of dopaminergic markers and Vglut2 in mouse mesodiencephalic dopaminergic nuclei A8-A13. J. Comp. Neurol. *529*, 1273–1292. https://doi.org/10.1002/cne.25020.

66. Mathis, A., Mamidanna, P., Cury, K.M., Abe, T., Murthy, V.N., Mathis, M.W., and Bethge, M. (2018). DeepLabCut: markerless pose estimation of user-defined body parts with deep learning. Nat. Neurosci. *21*, 1281–1289. https://doi.org/10.1038/s41593-018-0209-y.

67. Nath, T., Mathis, A., Chen, A.C., Patel, A., Bethge, M., and Mathis, M.W. (2019). Using DeepLabCut for 3D markerless pose estimation across species and behaviors. Nat. Protoc. *14*, 2152–2176. https://doi.org/10.1038/s41596-019-0176-0.

68. Hausmann, S.B., Vargas, A.M., Mathis, A., and Mathis, M.W. (2021). Measuring and modeling the motor system with machine learning. Curr. Opin. Neurobiol. *70*, 11–23. https://doi.org/10.1016/j.conb.2021.04.004.

69. Garland, T., Gleeson, T.T., Aronovitz, B.A., Richardson, C.S., and Dohm, M.R. (1995). Maximal sprint speeds and muscle fiber composition of wild and laboratory house mice. Physiol. Behav. *58*, 869–876. https://doi.org/10.1016/0031-9384(95)00148-c.

70. Leblond, H., L'Esperance, M., Orsal, D., and Rossignol, S. (2003). Treadmill locomotion in the intact and spinal mouse. J. Neurosci. *23*, 11411–11419. https://doi.org/10.1523/JNEUROSCI.23-36-11411.2003.

71. He, K., Zhang, X., Ren, S., and Sun, J. (2016). Deep Residual Learning for Image Recognition. In 2016 IEEE Conference on Computer Vision and Pattern Recognition (CVPR), pp. 770–778. https://doi.org/10.1109/CVPR.2016.90.

72. Insafutdinov, E., Pishchulin, L., Andres, B., Andriluka, M., and Schiele, B. (2016). DeeperCut: A Deeper, Stronger, and Faster Multi-Person Pose Estimation Model. Preprint at arXiv. https://doi.org/10.48550/arXiv.1605.03170.

73. Franklin, K., and Paxinos, G. (2008). The Mouse Brain in Stereotaxic Coordinates, 3rd Edition, p. Compact.

CellPress
OPEN ACCESS

STAR★METHODS

KEY RESOURCES TABLE

REAGENT or RESOURCE	SOURCE	IDENTIFIER
**Antibodies**		
goat anti-choline acetyltransferase	Sigma	AB144P, lots 3018862, 3675895, RRID: AB_2079751
donkey anti-goat Alexa 594	Invitrogen	Invitrogen A11058, lots 1975275, 2306782 RRID: AB_2534105
**Bacterial and virus strains**		
AAV2/retro-EF1a-DIOhChR2(H134R)-EYFP	Canadian Neurophotonics Platform Viral Vector Core Facility	RRID:SCR_016477
**Critical commercial assays**		
Advanced Cell Diagnostics RNAscope Multiplex Fluorescent Reagent Kit v2	ACD	323100-USM (no RRID)
**Experimental models: Organisms/strains**		
D1-cre mice: B6.FVB(Cg)-Tg(Drd1-cre)EY262Gsat/Mmucd	MMRRC	030989-UCD
D2-cre mice: B6.FVB(Cg)-Tg(Drd2-cre)ER44Gsat/Mmucd	MMRRC	032108-UCD-HEMI-M
**Oligonucleotides**		
RNAscope probe against Cre-C1	ACD	322381, lot 23081A
RNAscope probe against D_1 receptor-C4	ACD	406491-C4, lot 23080B
RNAscope probe against D_2 receptor-C4	ACD	406501-C4, lot 23080B
RNAscope probe against Vglut2-C4	ACD	319171-C4, lot 210498
RNAscope probe against VGAT-C4	ACD	319191-C4, lot 21251E
RNAscope probe against ChAT-C4	ACD	ACD 408731-C4, lot 21251E
RNAscope probe against D_1 receptor-C2	ACD	406491-C2, lot 213148
RNAscope probe against D_2 receptor-C3	ACD	406501-C3, lot 221658
**Software and algorithms**		
StereoInvestigator	MBF Bioscience	Version 1.1
Photoshop	Adobe	CS6
SigmaPlot	Systat	Version 12.0
StereoDrive	Neurostar	Version 3.3.3
DeepLabCut	mackenziemathislab.org	Versions 2.1.5.2 and 2.3.1
Norpix Streampix	1st Vision	Version 8
MATLAB	Mathworks	Version 2019a

RESOURCE AVAILABILITY

Lead contact

Further information and requests for resources and reagents should be directed to and will be fulfilled by the lead contact, Dimitri Ryczko (dimitri.ryczko@gmail.com).

Materials availability

This study did not generate new unique reagents.

Data and code availability

- All data reported in this paper will be shared by the lead contact upon request.
- This paper does not report original code.
- Any additional information required to reanalyze the data reported in this work paper is available from the lead contact upon request.

EXPERIMENTAL MODEL AND STUDY PARTICIPANT DETAILS

Ethics statement

All procedures were in accordance with the guidelines of the Canadian Council on Animal Care and were approved by the animal care and use committees of the Université de Sherbrooke (QC, Canada). Care was taken to minimize the number of animals used and their suffering.

Animals

We used D_1-cre knock-in mice (MMRRC repository stock number 030989-UCD, B6.FVB(Cg)-Tg(Drd1-cre)EY262Gsat/Mmucd) and D_2-cre knock-in mice (MMRRC repository stock number 032108-UCD-HEMI-M, B6.FVB(Cg)-Tg(Drd2-cre)ER44Gsat/Mmucd).[16,17] Heterozygous mice were used, and mice were genotyped as previously described.[20,21,65] Animals had *ad libitum* access to food and water, with lights on from 6 a.m. to 8 p.m. D_1-cre mice used for *in vivo* experiments were 9–45 weeks old at time of use (6 males, 3 females). D_2-cre mice used for *in vivo* experiments were 8–30 weeks old at time of use (9 males, 5 females). Mice used for RNA-scope experiments were 10–17 weeks old (D_1-cre: 1 female, 2 males; D_2-cre: 2 females, 1 male; wild-type: 2 females, 4 males, 2 undetermined).

METHOD DETAILS

Virus injection and optic fiber implantation

The procedure was adapted from previously reported ones.[11,20,21] Briefly, mice were anesthetized using isoflurane (induction: 5%, 500 mL/min; maintenance: 1.5–2.5%, 100 mL/min) delivered with a SomnoSuite (Kent Scientific, Torrington, CT, USA). Mice were placed in a Robot Stereotaxic instrument coupled with StereoDrive software (Neurostar, Tübingen, Germany). Mice were given buprenorphine as an analgesic (0.1 mg/kg s.c., volume 0.3 mL). An incision was made on the scalp, a hole was drilled in the cranium and a 10 μL Hamilton syringe locked into the Robot Stereotaxic instrument was used to inject unilaterally in CnF or in PPN a volume of 300 nL[18] of a solution containing an adeno-associated virus (AAV) driving the expression of the light-activated cation channel channelrhodopsin fused with a yellow fluorescent protein in a cre-dependent manner (AAV2/retro-EF1a-DIOhChR2(H134R)-EYFP, titer 9.8×10^{12} particles per milliliter, Canadian Neurophotonics Platform Viral Vector Core Facility, RRID:SCR_016477). The AAV solution was injected unilaterally into the CnF (anteroposterior −4.80 to −4.85 mm, mediolateral +1.10 to +1.15 mm, dorsoventral −2.90 mm relative to bregma) or the PPN (anteroposterior −4.65 to −4.72 mm, mediolateral +1.20 mm, dorsoventral −3.75 mm relative to bregma) at a rate of 0.05 μL/min. The syringe was left in place for 1 min before being removed. Then, an optic fiber (200 μm core, 0.22 NA, 5 mm length, Thorlabs, Newton, NJ, USA) held in a ceramic or stainless-steel ferrule was placed 500 μm above the right CnF at −4.80 to −4.85 mm anteroposterior, +1.10 to +1.15 mm mediolateral, −2.40 mm dorsoventral or right PPN at −4.65 to −4.72 mm anteroposterior, +1.20 mm mediolateral, −3.35 mm dorsoventral relative to bregma.[20,21] The ferrule was secured on the cranium using two 00-96×1/16 mounting screws (HRS Scientific, QC, Canada) and dental cement (A-M Systems, Sequim, WA, USA).

In vivo optogenetic stimulation

The procedure was as previously reported.[20,21] Briefly, the implanted optic fiber was connected to a 470 nm laser (Ikecool, Anaheim, CA, USA) using a pigtail rotary joint (Thorlabs). The laser was driven with a Grass S88X to generate the stimulation trains (2 or 10 s trains, 10 ms pulses, 20 Hz[18–21]). To visualize optogenetic stimulation, a copy of the stimulation trains was sent to a small (diameter 0.5 cm) low-power (0.13 W) red LED coupled with a 120 MΩ resistance.[20,21] The LED was placed in the field of view of the camera placed above the open field. The 470 nm laser was adjusted to 4.0–44.0% of laser power. The corresponding power measured at the fiber tip with a power meter (PM100USB, Thorlabs) was 0.06–28.60 mW. To determine whether evoked locomotor responses were specific to blue light we replaced the blue laser by a red one (589 nm) as we did in a previous study.[20] The 589 nm laser was adjusted to 55% of laser power. The corresponding power measured at the fiber tip with a power meter (PM100USB coupled with a S120C power sensor, Thorlabs) was 4.50 mW. Estimation of the loss of light irradiance as a function of distance from the optic fiber tip is based on calculations made in brain tissue (https://web.stanford.edu/group/dlab/cgi-bin/graph/chart.php.[59]). Such estimations were previously used by us[21] and others.[19,27] The light cone angle (18.6°) emanating from the optic fiber was estimated based on fiber numerical aperture (0.22) and refractive index of brain tissue (1.36).[58]

Open-field locomotion

The procedure was as previously reported.[20,21] Briefly, locomotor activity was filmed from above in a 40 × 40 cm open field arena at 30 fps using a Canon Vixia HF R800 camera. To measure the effects of optogenetic stimulation, locomotor activity was recorded during trials of 15 min during which 10 s stimulation trains were delivered every 80 s at various laser powers. In some experiments, 2 s stimulation trains were applied during ongoing locomotor bouts occurring spontaneously in the open field arena with at least 80 s between stimulations. Video recordings were analyzed with DeepLabCut to track user-defined body parts[66–68] and a custom MATLAB script (Mathworks, Natick, MA, USA).[20,21] We tracked the body center positions, the corners of the arena for distance calibration, and the small LED to detect optogenetic stimulations. Timestamps were extracted using Video Frame Time Stamps (MATLAB File Exchange). Body center positions and timestamps were used to calculate locomotor speed. Body center and tail

base positions were excluded if their detection likelihood by DeepLabCut was <0.8, if they were outside of the open-field arena, or if body center speed exceeded the maximum locomotor speed recorded in mice (334 cm/s[69]).

To estimate angular velocity of the animal as a function of time in the open field arena, a body orientation vector was calculated in each frame using the positions of the tail base and body center that were detected using DeepLabCut. Using two body orientation vectors obtained from two consecutive frames, we identified the unique circle that was tangent to both vectors at the level of the body center positions (Figure 3O). The angular displacement of the animal was then defined as the angle between the two body centers and the center of the circle, which is equal to the angle between the two orientation vectors (θ, Figure 3O). We could therefore calculate the angular velocity by measuring the angle between two consecutive body orientation vectors over time. Angular velocity was positive when the animal was turning toward its right side.

Changes in body orientation angle were evaluated as in Usseglio and colleagues.[22] The body orientation angle α (Figures 5B and 6B) between the x axis of the open field and the body axis vector drawn from the tail base (T) to the middle (EM) of a line drawn between left ear (EL) and right ear (ER) was calculated for each frame. To pool data from multiple trials, this angle was normalized to the average angle during the 500 ms preceding photoactivation. Angles increasing clockwise (i.e., toward the stimulated side) were considered positive. Changes in head rotation angle were evaluated as in Usseglio and colleagues (2020).[22] The head rotation angle β (Figures 5F and 6F) between the body axis vector (body axis vector drawn from the tail base [T] to the middle [EM] of a line drawn between left ear [EL] and right ear [ER]) and a line perpendicular to the vector drawn from left ear (EL) to right ear (ER) was calculated for each frame. To pool data from multiple trials, this angle was normalized to the average angle during the 500 ms preceding photoactivation. Angles increasing clockwise (i.e., toward the stimulated side) were considered positive.

Linear corridor and limb kinematics

This test was used to record limb kinematics and paw movements as previously reported.[20,21] Briefly, to label hindlimb joints, mice were anesthetized, the hindlimb was shaved and ~2 mm white dots were drawn on the iliac crest, hip, knee, ankle, and metatarsophalangeal (MTP) joints, and the toe tip using a fine-tip, oil-based paint marker (Sharpie). For paw tracking, no labeling of paw underside was needed. After 20 min of recovery from anesthesia, mice were placed in a 1 m long, 8 cm wide transparent corridor. Hindlimb kinematics and paw movements were recorded at 300 fps using two high-speed Genie Nano M800 cameras (Teledyne DALSA, Waterloo, ON, Canada) coupled to a computer equipped with Norpix Streampix software (1st Vision, Andover, MA, USA). Hindlimb kinematics were recorded from the side and paw movements were recorded from below with a camera placed on the side that was directed to a 45-degree mirror placed below the corridor.[20,21] For distance calibration, 4 markers (diameter 0.5 cm) were distributed 5 cm apart in the field of view of each camera. To detect optogenetic stimulation, a LED that received a copy of the stimulation trains was placed in the field of view of both cameras. Animals were recorded during optogenetic-evoked locomotion and during spontaneous locomotion evoked by a gentle touch of the animal's tail or a gentle air puff generated by a small air bulb.

For hindlimb kinematics, the positions of the joints and toe tip were detected using DeepLabCut. A moving average of the MTP speed was used to determine the stance and swing phases by detecting the touchdown and lift-off times with a speed threshold of 15 cm/s, and a minimum of 14 frames above threshold for the lift-off detection.[20,21] The joint positions were used to extract the angles of the hip, knee, ankle, and MTP joints. The angular excursions as a function of time were normalized to step cycle duration using MTP touchdown times as a reference.[20,21,70] For paw movements, videos recorded from below were used to track the position of the MTPs of the four paws with DeepLabCut. Paw speeds were calculated and smoothened with a moving average (on five frames) using a custom MATLAB script. Frames were excluded from the analysis if the MTPs or any limb joints or the toe tip had a detection likelihood by DeepLabCut was <0.8, if any paw's or joint's speed exceeded 400 cm/s (i.e., maximum locomotor speed of a mouse with a 20% margin to account for increased speed of individual body parts), or if the distance between two adjacent joints was >2.3 cm (i.e., length of the tibia in wild-type mice).[20,21]

In some experiments, the linear corridor was used to record the effects of optogenetic stimulation with blue light at various laser powers in the PPN of D_2-cre mice (stimulation train of 10 s, 10 ms pulses, 20 Hz) or in the CnF in D_2-cre mice during ongoing locomotion (stimulation train of 2 s, 10 ms pulses, 20 Hz).

To analyze limb coordination during evoked motor arrests in the linear corridor, we used video recordings of paw movements filmed from below coupled with an analysis inspired by Goñi-Erro and colleagues (2023).[23] Briefly, using the detected paw positions, we attributed phase values to the position of each limb pair, based on the distance between left and right paws along the corridor length axis. A phase of +180° corresponds to maximum positive alternation, 0° corresponds to a symmetrical position of both limbs on a line perpendicular to the corridor length axis, and −180° corresponds to maximum negative alternation (Figure S6D). These measurements assume that the animals move along the corridor length in a straight line.

To determine the time of arrest and resume, we smoothed the phase data with a moving average over 10 frames and calculated the phase variation from frame to frame. Latency to resume was calculated for each trial as the time difference between light onset and the first frame after light onset where limb-pair phase variation was below 0.1°/frame within a sequence of twenty contiguous frames with variation below 0.1°/frame. Latency to resume was calculated for each trial as the time difference between light offset and the first frame after light offset where phase variation was above 0.1°/frame within a sequence of twenty contiguous frames with variation above 0.1°/frame.

To determine whether limb movement resumes from the same position after the arrest, we measured the phase difference by subtracting the phase measured at movement arrest from the phase measured at movement resume for each limb pair.

To determine whether a new step cycle was initiated at movement resume, we evaluated “step cycle continuity” in a way inspired by Goñi-Erro and colleagues (2023)[23] by comparing the sign of limb-pair phase slopes before arrest and after movement resume (Figures S6I and S6J). Peaks and troughs were detected using islocalmax and islocalmin functions in MATLAB with a minimum prominence of 60° in both cases. To define the sign of the slopes at arrest and resume, we considered the last peak or trough before arrest time point, and the first one after resume time point. Continuity assumes that if at motor arrest the phase was e.g., ascending (positive slope), then at movement resume, phase should continue to have a positive slope. In cases when at motor arrest the phase had peaked (slope almost equal to zero), then at movement resume the slope sign should switch. For forelimb pairs and hindlimb pairs we quantified continuity either as false, true, or not detected either when no time of resume was detected, or when no peak or trough was detected after time of resume.

DeepLabCut networks

Some of the networks used were the same as those previously described.[20,21] Briefly, for the analysis of locomotion in the open-field arena, we labeled the body center, the tail base, the corners of the arena, and the LED to visualize optogenetic stimulation. We used a ResNet-50-based neural network[71,72] with default parameters for 1,030,000 training iterations. We validated with one shuffle and found that the test error was 2.28 pixels and the train error 1.85 pixels.[20,21]

For limb kinematics analysis, we labeled the 5 joints and the toe tip, four distance calibration markers, and the low-power LED to visualize optogenetic stimulation. We used a ResNet-50-based neural network[71,72] with default parameters for 1,030,000 training iterations and one refinement of 1,030,000 iterations. We validated with one shuffle and found that the test error was 2.03 pixels and the train error 1.87 pixels.[20,21]

For the analysis of body orientation and head rotation, we labeled the body center, the two ears, the tail base, the corners of the arena, and the LED to visualize optogenetic stimulation. We used a ResNet-50–based neural network,[71,72] with default parameters for 1,030,000 training iterations and one refinement of 1,030,000 iterations. We validated with one shuffle and found that the test error was 2.78 pixels and the train error 2.04 pixels.

For the analysis of paw movements from below in the linear corridor, we labeled the four paws, four distance calibration markers, and the low-power LED to visualize optogenetic stimulation. We used a ResNet-50–based neural network,[71,72] with default parameters for 1,030,000 training iterations. We validated with one shuffle and found that the test error was 2.26 pixels and the train error 1.74 pixels.

Treadmill

Mice were placed on a treadmill and locomotor activity was filmed from the side in a 37.5 × 5.1 cm motorized treadmill (Model 8709, Letica Scientific Instruments, Panlab, Spain) arena at 30 fps using a Canon Vixia HF R800 camera. To measure the effects of optogenetic stimulation, the treadmill was switched on during trials of around 30 s (speed 12 cm/s) during which a stimulation train (2s, 10 ms pulses, 20 Hz) was delivered.

Histology

Procedures were as previously reported.[20,21,65] Briefly, mice were anesthetized using isoflurane (5%, 2.5 L per minute) and transcardially perfused with 30–50 mL of a phosphate buffer solution (0.1M) containing 0.9% of NaCl (PBS, pH = 7.4), followed by 50 mL of PBS solution containing 4% (w/v) of paraformaldehyde (PFA 4%). Post-fixation of the brains was performed for 24 h in a solution of PFA 4%. Brains were incubated in a PB solution containing 20% (w/v) sucrose for 24 h before histology. Brains were snap frozen in methylbutane (−45°C ± 5°C) and sectioned at −20°C in 40 μm-thick coronal slices using a cryostat (Leica CM 1860 UV). Floating sections at the level of the MLR were collected under a Stemi 305 stereomicroscope (Zeiss) and identified using the mouse brain atlas of Franklin and Paxinos (2008).[73]

Immunofluorescence

The procedure was as previously reported.[20,21,65] All steps were carried out at room temperature unless stated otherwise. The sections were rinsed three times during 10 min in PBS and incubated during 1h in a blocking solution containing 5% (v/v) of normal donkey serum and 0.3% Triton X-100 in PBS. The sections were incubated during 48 h at 4°C in a blocking solution containing the primary antibody against ChAT (goat anti-choline acetyltransferase, Sigma AB144P, lots 3018862, 3675895 (1:100), RRID: AB_2079751) and gently agitated with an orbital shaker. Then, the sections were washed three times in PBS and incubated during 4 h in a blocking solution containing a secondary antibody to reveal ChAT (donkey anti-goat Alexa 594, Invitrogen A11058, lots 1975275, 2306782 (1:400), RRID: AB_2534105). The slices were rinsed three times in PBS for 10 min and mounted on Colorfrost Plus slides (Fisher 1255017) with a medium with DAPI (Vectashield H-1200) or without DAPI (Vectashield H-1000), covered with a 1.5 type glass coverslip and stored at 4°C before observation.

RNAscope

The procedure was as previously reported.[21] To detect Vglut2 (Slc17a6), VGAT (Slc32a1), ChAT, Cre, D_1 receptor and D_2 receptor mRNAs in coronal brain slices, we used the Advanced Cell Diagnostics RNAscope Multiplex Fluorescent Reagent Kit v2 Assay on fixed-frozen tissue samples (ACD 323100-USM). All steps were carried out at room temperature unless stated otherwise. Mice

were anesthetized with isoflurane (5%, 2.5 L per minute) and transcardially perfused with 50 mL of PBS, followed by 50 mL of PBS solution containing 4% (w/v) of PFA. Post-fixation of the brains was performed for 24 h in a solution of PFA 4%. Brains were incubated in a PB solution containing 20% (w/v) sucrose for 24 h before being snap-frozen on dry ice. Brains were then sectioned at −20°C in 15 μm-thick coronal slices using a cryostat (Leica CM 1860 UV) and mounted onto Colorfrost Plus glass slides (Fisher 1255017). Sections were air-dried during 2 h at −20°C, washed in PBS during 5 min, and baked during 30 min at 60°C in an HybEZ II oven (ACD 321721). Sections were then dehydrated in increasing concentrations of ethanol (50%, 70%, 100%, 100%, 5 min each), treated with hydrogen peroxide during 10 min (ACD 322381, lots 2011534, 2016054), with RNAscope 1X Target Retrieval Agent during 5 min (ACS 322000, lots 2011356, 2013297), and with protease III during 30 min in the oven at 40°C (ACD 322381, lot 2011534). Sections were then treated during 2 h at 40°C in the oven with either i) an RNA hybridization antisense probe against Cre-C1 (ACD, 312281, lot 23081A) and another against mouse D_1 receptor-C4 (ACD 406491-C4, lot 23080B) or against D_2 receptor-C4 (ACD 406501-C4, lot 23080B); or ii) a probe against Vglut2-C4 (ACD 319171-C4, lot 210498) or VGAT-C4 (ACD 319191-C4, lot 21251E) or ChAT-C4 (ACD 408731-C4, lot 21251E), and another against D_1 receptor-C2 (ACD 406491-C2, lot 213148) or D_2 receptor-C3 (ACD 406501-C3, lot 221658). After overnight incubation in a saline sodium citrate solution (175.3g of NaCl and 88.2g of sodium citrate in 1L of distilled water, pH = 7.0), C1, C2, C3 probe amplification was done using RNAscope Multiplex Fluorescent Detection Reagents v2 (ACD 323110, lots 2011351, 2014260). C4 probe amplification was done with the RNAscope Multiplex Fluorescent Detection Reagents v2, with the addition of the HRP-C4 (ACD 323121, lots 2011711, 2013504). C1 and D_2 receptor-C3 probes were revealed using Opal Dye 520 (Akoya Biosciences FP1487001KT, lot 201008031, 1:1500) and all other C2, C3 and C4 probes were revealed using Opal Dye 690 (Akoya Biosciences FP1497001KT lot 201008030, 1:1500). Sections were counterstained with DAPI (ACD 323108, lots 2011350, 2014268), mounted with a ProLong Gold Antifade Mountant (Invitrogen P36930, lots 2305164, 2006594), covered with a 1.5 type glass coverslip and stored at +4°C before observation.

Microscopy

Brain sections were observed using a Zeiss AxioImager M2 microscope bundled with StereoInvestigator 2018 software (v1.1, MBF Bioscience). To show the expression of ChR2-EYFP in the CnF or PPN, high magnification (60X) photographs were taken using a Confocal FV1000-IX81 (Olympus). Composite images were assembled, and the levels were uniformly adjusted in Photoshop CS6 (Adobe) to make all fluorophores visible and avoid pixel saturation, and digital images were merged.

Cell counting

The procedure was as previously reported.[21,65] To estimate the number of mRNA-positive cells, for each animal one to three coronal brain slices were photographed at 40X magnification with an epifluorescent microscope Zeiss AxioImager M2. A region of interest was identified based on the mouse brain atlas and the cells positive for D_1 receptor, D_2 receptor, Cre, Vglut2, VGAT or ChAT mRNAs were counted. Our criterion for cell count was a labeling of the cell body as previously reported.[21,65]

QUANTIFICATION AND STATISTICAL ANALYSIS

Data are presented as mean ± standard error of the mean (SEM) unless stated otherwise. No statistical method was used to predetermine sample sizes, which are similar to those used in the field (e.g.,[18,20]). Statistical details of experiments can be found in the figure legends. No randomization or blinding procedure was used. Statistical analysis was done using Sigma Plot 12.0. Parametric analyses were used when assumptions for normality and equal variance were respected, otherwise non-parametric analyses were used. Normality was assessed using the Shapiro-Wilk test. Equal variance was assessed using the Levene test. To compare the means between two dependent groups, a parametric two-tailed paired t test or a non-parametric Wilcoxon test was used. To compare the means between two independent groups, a two-tailed t test or a non-parametric Mann-Whitney rank-sum test was used. For more than two dependent groups, a parametric one-way analysis of variance (ANOVA) for repeated measures or a non-parametric Friedman ANOVA for repeated measures on ranks was used. ANOVAs were followed by a Student Newman-Keuls post hoc test for multiple comparisons between groups. Sigmoidal regressions between variables, their significance, and the 95% confidence intervals were calculated using Sigma Plot 12.0. Statistical differences were assumed to be significant when $p < 0.05$.

Cell Reports, Volume *43*

Supplemental information

Dopamine-sensitive neurons

in the mesencephalic locomotor region

control locomotion initiation, stop, and turns

Andrea Juárez Tello, Cornelis Immanuel van der Zouwen, Léonie Dejas, Juan Duque-Yate, Joël Boutin, Katherine Medina-Ortiz, Jacinthlyn Sylvia Suresh, Jordan Swiegers, Philippe Sarret, and Dimitri Ryczko

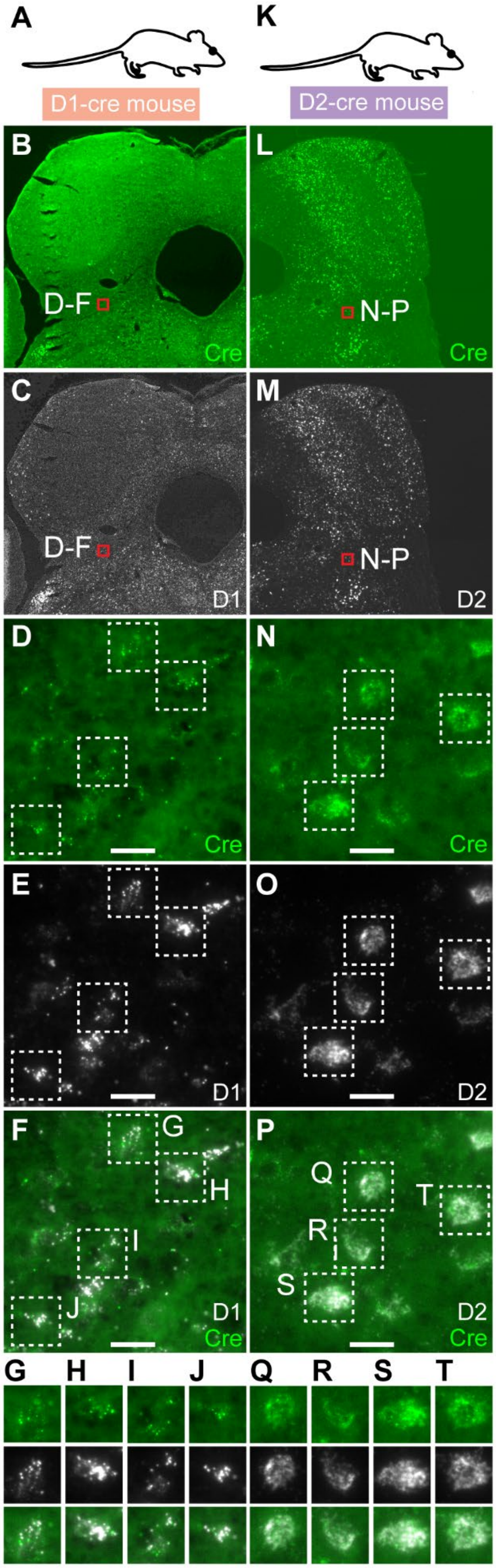

Figure S1. Expression of *cre mRNA* in D_1-cre and D_2-cre mice in the Mesencephalic Locomotor Region. Related to Figure 2. (A-F) Photomicrographs from a D_1-cre mouse illustrating example cells expressing both *cre mRNA* (green, B,D,F) and *D_1 receptor mRNA* (white, C,E,F). D-F illustrate magnifications of the region delineated in red in B-C. Cell count in 3 D_1-cre mice indicated that 86% of MLR neurons positive for *cre mRNA* expressed *D_1 receptor mRNA* (17/19 *cre mRNA*$^+$ cells in mouse 1, 26/32 in mouse 2, 25/29 in mouse 3, 1 to 3 slices per mouse). **(G-J)** Single cells extracted from the dashed squares in D-F. **(K-P)** Photomicrographs from a D_2-cre mouse illustrating example cells showing expression of *cre mRNA* (green, L,N,P) and *D_2 receptor mRNA* (white, M,O,P). N-P illustrate magnifications of the region delineated in red in L-M. Cell count in 3 D_2-cre mice indicated that 90% of MLR neurons positive for *cre mRNA* expressed *D_2 receptor mRNA* (17/17 *cre mRNA*$^+$ cells in mouse 1, 26/32 in mouse 2, 24/27 in mouse 3; 1 to 3 slices per mouse). **(Q-T)** Single cells extracted from the dashed squares in N-P. Scale bars, 20 μm.

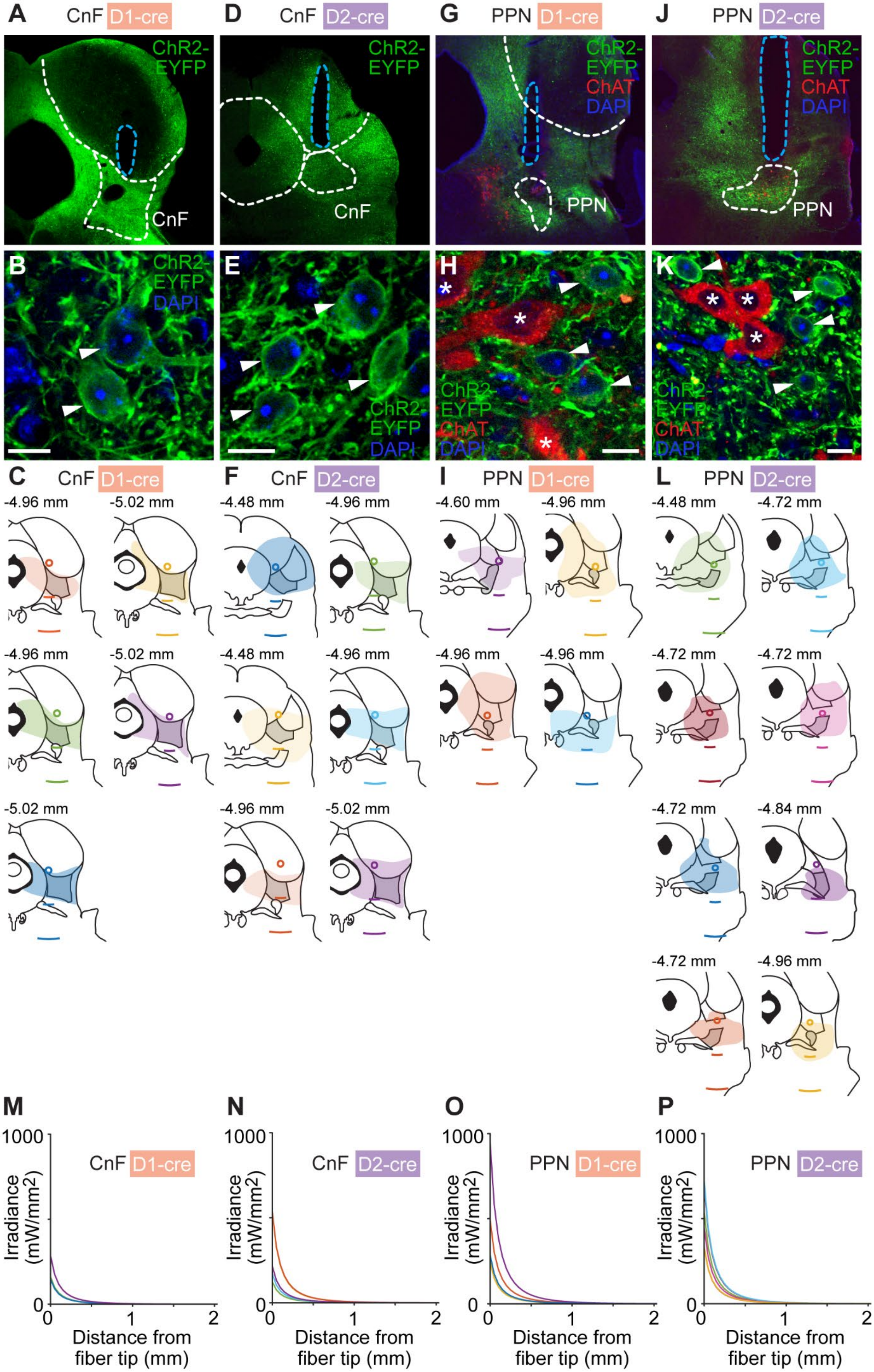
A
CnF D1-cre
ChR2-EYFP
CnF
D
CnF D2-cre
ChR2-EYFP
CnF
G
PPN D1-cre
ChR2-EYFP
ChAT
DAPI
PPN
J
PPN D2-cre
ChR2-EYFP
ChAT
DAPI
PPN
B
ChR2-EYFP
DAPI
E
ChR2-EYFP
DAPI
H
ChR2-EYFP
ChAT
DAPI
K
ChR2-EYFP
ChAT
DAPI
C
CnF D1-cre
-4.96 mm
-5.02 mm
-4.96 mm
-5.02 mm
-5.02 mm
F
CnF D2-cre
-4.48 mm
-4.96 mm
-4.48 mm
-4.96 mm
-4.96 mm
-5.02 mm
I
PPN D1-cre
-4.60 mm
-4.96 mm
-4.96 mm
-4.96 mm
L
PPN D2-cre
-4.48 mm
-4.72 mm
-4.72 mm
-4.72 mm
-4.72 mm
-4.84 mm
-4.72 mm
-4.96 mm
M
CnF D1-cre
N
CnF D2-cre
O
PPN D1-cre
P
PPN D2-cre
Irradiance (mW/mm2)
1000
0
Distance from fiber tip (mm)
0
1
2

Figure S2. Adeno-associated virus injections and optic fiber implantations. Related to Figure 2. **(A,D,G,J)** Photomicrographs showing the position of cells infected by virus injection (green) in the cuneiform nucleus (CnF) or pedunculopontine nucleus (PPN) and position of the optic fiber right above the CnF (A,D) or PPN (G,J) in D_1-cre mice (A,G) or D_2-cre mice (D,J). In G and J, cells immunoreactive for choline acetyltransferase (ChAT) are shown in red, the nuclear marker DAPI is shown in blue. **(B,E,H,K)** Confocal magnifications of the CnF or PPN regions delineated in A,D,G,J, showing example cells (arrowheads) expressing channelrhodopsin fused with enhanced yellow fluorescent protein (ChR2-EYFP, green) driven by the adeno-associated virus in a cre-dependent manner. In H and K, cells immunoreactive for ChAT are shown in red (asterisks) and the nuclear marker DAPI is shown in blue. Scale bars, 10 μm. **(C,F,I,L)** Histological locations of the cells infected by the adeno-associated virus and of the tips of the optic fibers. On each slice, the 18.6° arcs illustrate the estimated cones of irradiance at 1 mm and 2 mm away from optic fiber tip for the maximal laser intensity used for each mouse. The cone apex angle was defined based on measurements done in brain tissue (see STAR Methods). **(M-P)** For each animal group, estimated decrease of irradiance as a function of distance from optic fiber tip based on measurements done in brain tissue (see STAR Methods). The colors for each animal are the same as in C,F,I,L. Note that in some mice the same maximal laser intensity was used, causing trace overlap here.

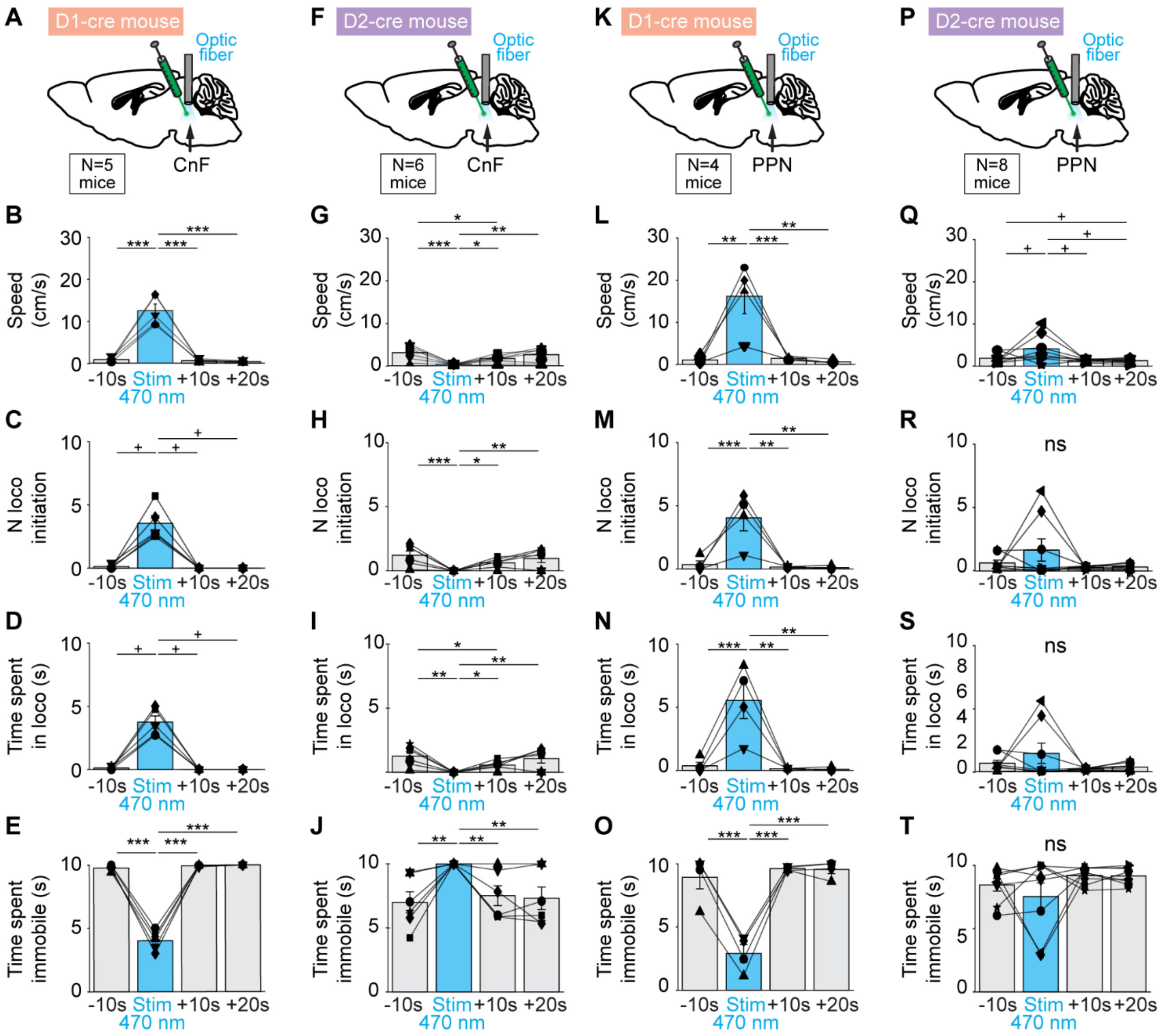

Figure S3. Effect of optogenetic activation with blue light (470 nm) of MLR dopamine-sensitive neurons on locomotor parameters. Related to Figure 2. (A-T) Evolution of locomotor parameters (mean ± sem) (see STAR Methods) before (-10 to 0 s), during (0 to 10 s), and after (10 to 20 s and 20 to 30 s) optogenetic stimulation with a blue laser (470 nm, 10 s train, 20 Hz, 10 ms pulses) of CnF in D_1-cre mice (9-15% of laser power) (A-E), CnF in D_2-cre mice (8-12% of laser power) (F-J), PPN in D_1-cre mice (10-24% of laser power) (K-O) and PPN in D_2-cre mice (10-31% of laser power) (P-T). *P<0.05, **P<0.01, ***P<0.001, Student–Newman–Keuls test after a one-way ANOVA for repeated measures (P<0.001 in B,E,G,H,N,O; P<0.01 in I,J,L,M; ns, not significant P>0.05 in T); +P<0.05, Student–Newman–Keuls test after a Friedman repeated measures ANOVA on ranks (P < 0.01 in C,D,Q); ns, not significant one-way ANOVA for repeated measures (P > 0.05 in R,S). When comparing CnF D_1, CnF D_2, PPN D_1 and PPN D_2 animal groups, locomotor speed during the 10 s before stimulation was not different between groups, except for the CnF D_2 group that displayed a higher speed than the CnF D_1 group (P < 0.05 Student Newman-Keuls after a One Way ANOVA, P < 0.05).

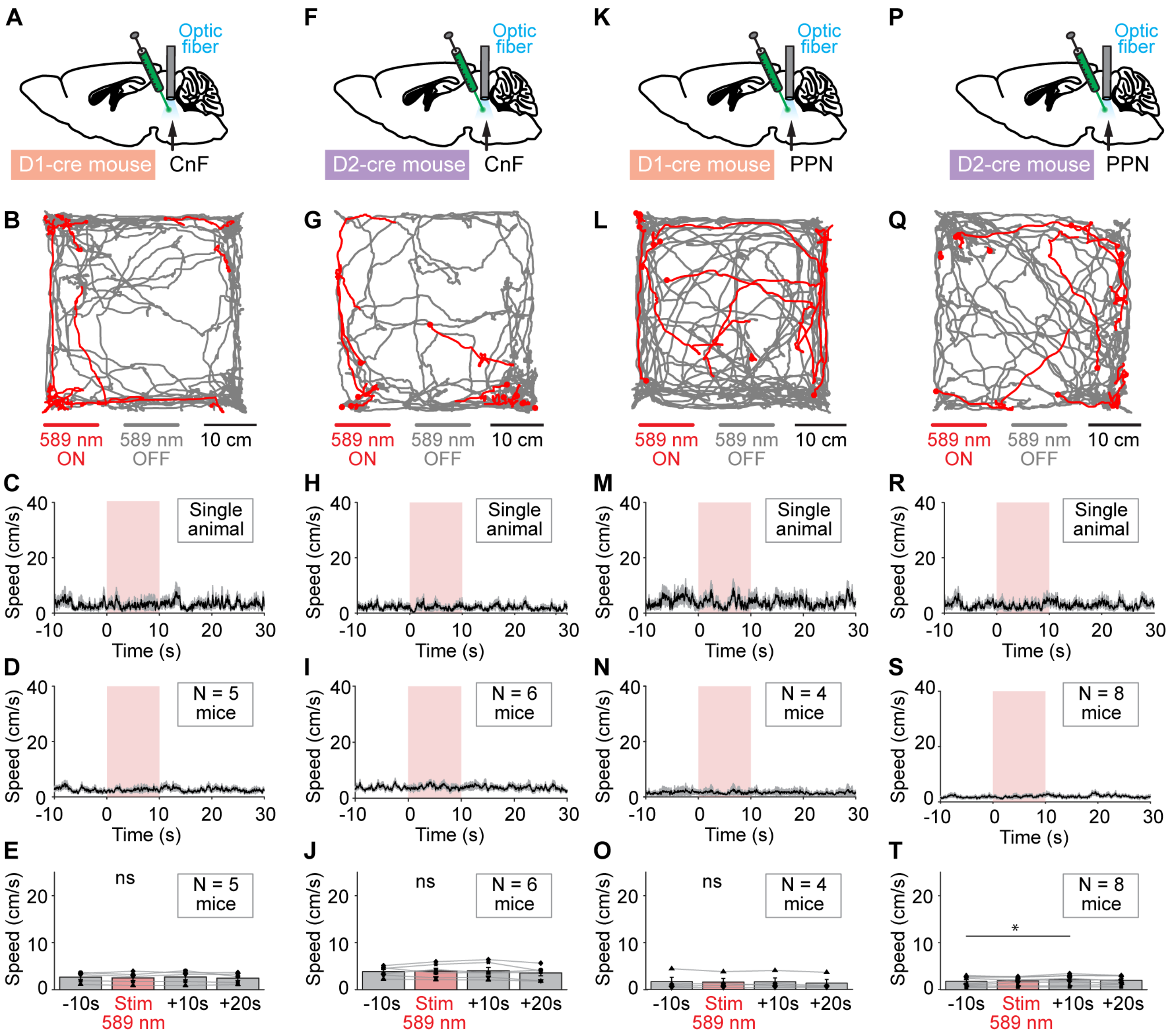

Figure S4. Effect of optogenetic activation with red light (589 nm) of MLR dopamine-sensitive neurons on locomotion. Related to Figure 2. (A-T) Locomotor activity and evolution of speed (mean ± sem) (see STAR Methods) before (-10 to 0 s), during (0 to 10 s), and after (10 to 20 s and 20 to 30 s) optogenetic stimulation with a red laser (589 nm, 10 s train, 20 Hz, 10 ms pulses, 55% of laser power) of CnF in D_1-cre mice (A-E), CnF in D_2-cre mice (F-J), PPN in D_1-cre mice (K-O) and PPN in D_2-cre mice (P-T). *$P<0.05$, Student–Newman–Keuls test after a one-way ANOVA for repeated measures ($P<0.05$ in T); ns, not significant one-way ANOVA for repeated measures ($P>0.05$ in E,J,O).

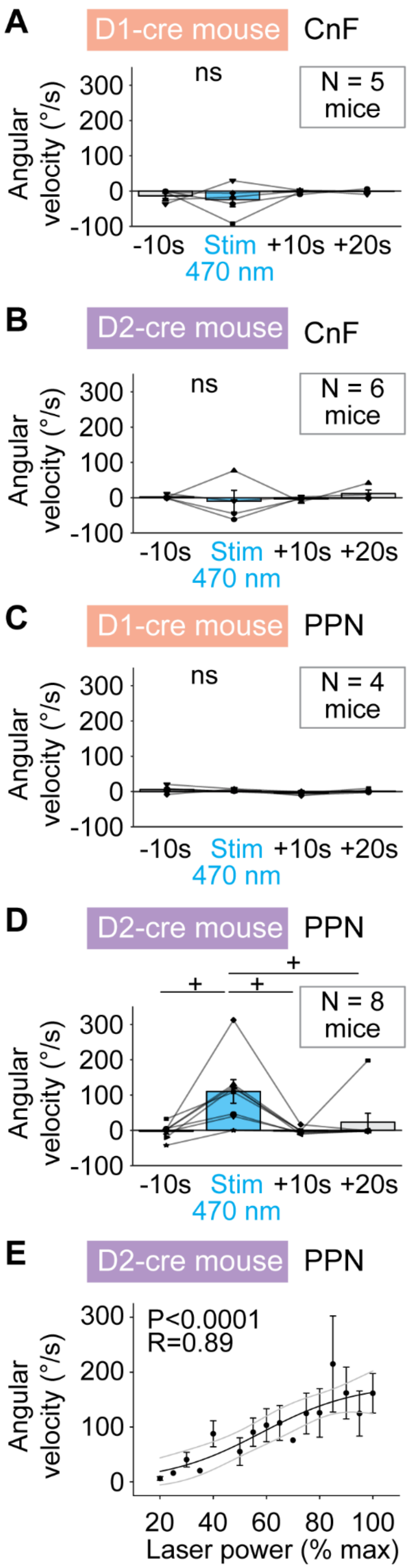

Figure S5. Effect of optogenetic activation with blue light (470 nm) of dopamine-sensitive neurons in the cuneiform nucleus (CnF) or pedunculopontine nucleus (PPN) on angular velocity. Related to Figure 3. (A-D) Evolution of angular velocity (mean ± sem) in the open field arena (see STAR Methods) before (-10 to 0 s), during (0 to 10 s), and after (10 to 20 s and 20 to 30 s) optogenetic stimulation with a blue laser (470 nm, 10 s train, 20 Hz, 10 ms pulses) of CnF in D_1-cre mice (9-15% of laser power) (A), CnF in D_2-cre mice (8-12% of laser power) (B), PPN in D_1-cre mice (10-24% of laser power) (C) and PPN in D_2-cre mice (10-31% of laser power) (D). $^+P<0.05$, Student–Newman–Keuls test after a Friedman repeated measures ANOVA on ranks ($P<0.05$); ns, not significant one-way ANOVA for repeated measures ($P>0.05$ in A-C). **(E)** Relationship between angular velocity (mean ± SEM) and laser power (470 nm, 10 s train, 20 Hz, 10 ms pulses, 4-34% of laser power, bin width 5%, 1 to 8 mice per bin) applied in the PPN of D_2-cre mice. The data followed a sigmoidal function (solid black line). The coefficient of correlation (R), its significance (P), and the confidence intervals (gray) are illustrated.

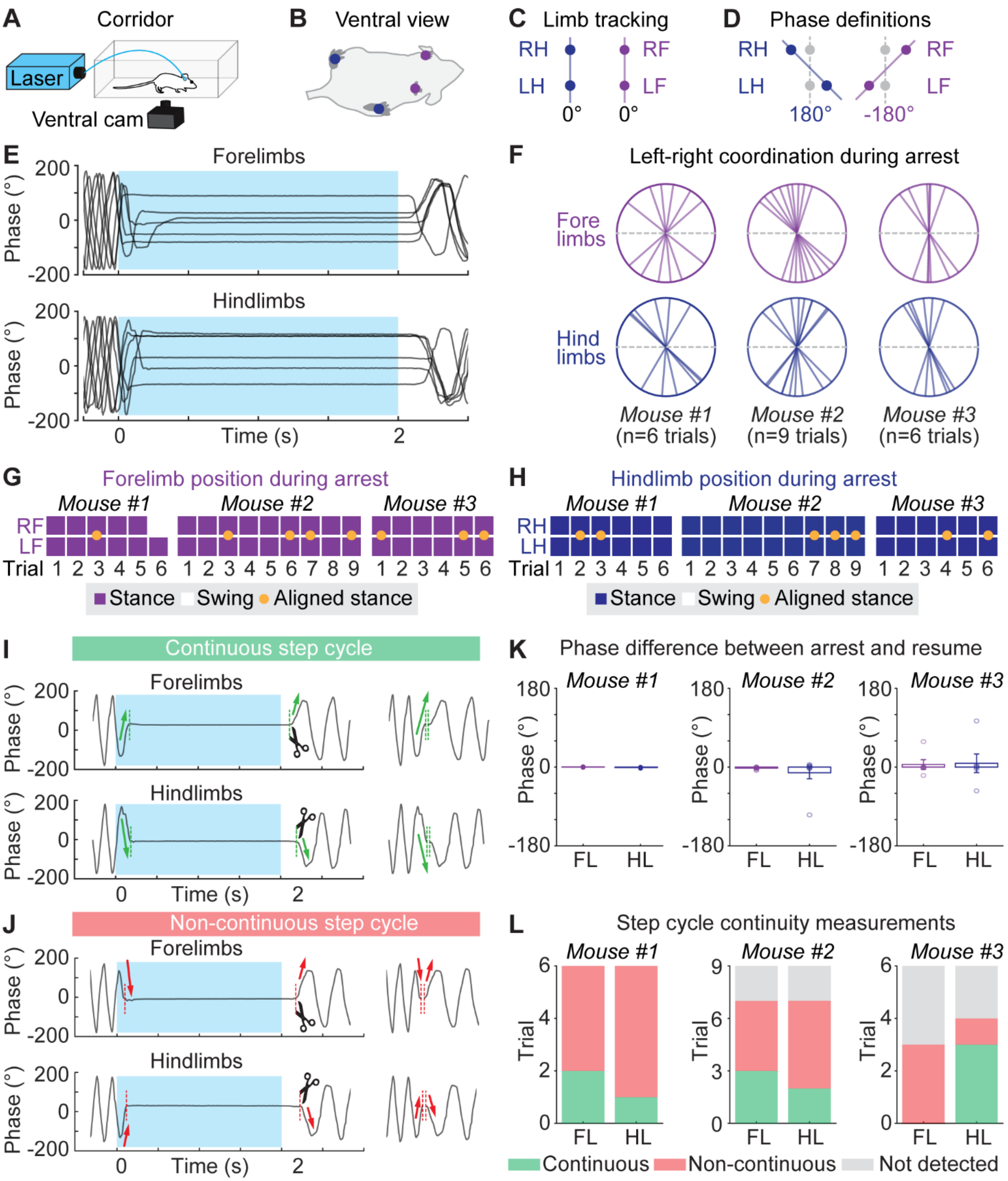

Figure S6. Coordination of limb movements during arrests evoked by CnF photostimulation in D_2-cre mice. Related to Figure 5. (A) Limb movements were filmed from below in a transparent linear corridor (see STAR Methods). **(B)** Forelimb and hindlimb paws were detected using DeepLabCut (see STAR Methods). **(C-D)** To analyze left-right forelimb coordination based on paw tracking, phase values were defined by the distance on the x axis (i.e. corridor length axis) between left and right forelimbs. The same analysis was carried out for hindlimbs. **(E)** Left-right coordination phase values for forelimbs (top) and hindlimbs (bottom) during locomotion temporarily interrupted by CnF photostimulation in an example D_2-cre mouse (n = 6 trials) (470 nm, 2 s train, 20 Hz, 10 ms pulses, 10% of laser power). **(F)** Left-right coordination for forelimbs (top) and hindlimbs (bottom) during arrest evoked CnF photostimulation in D_2-cre mice (n = 3 mice, 6-9 trials per mouse, 470 nm, 2 s train, 20 Hz, 10 ms pulses, 10-12% of laser power). For each trial, the line that unites the left and right forelimb paws, or

the left and right hindlimb paws is drawn in a circle. Dashed grey lines correspond to the corridor length axis. **(G-H)** Forelimb (purple) and hindlimb (blue) positions during arrest in the corridor for CnF stimulation trials in D_2-cre mice (n = 3 mice, 6-9 trials per mouse). Solid squares represent stance, and empty squares represent swing. Yellow dots illustrate that RH and LH, or RF and LF were aligned perpendicular (90 ± 15º) to the corridor length axis. **(I)** Representative trial illustrating continuity in the step cycle after light offset. **(J)** Representative trial illustrating non-continuity in the step cycle after light offset. **(K)** Phase difference (mean ± sem) between arrest time points and resume time points for forelimbs (FL) and hindlimbs (HL) in three mice (6-9 trials per mouse). **(L)** Step cycle continuities and non-continuities illustrated by arrows in I-J, quantified as a binary outcome for all trials for each of the three mice tested. Circles represent individual trials (n = 3 mice, 6-9 trials per mouse). Blue rectangles (E, I and J) illustrate photostimulation duration.

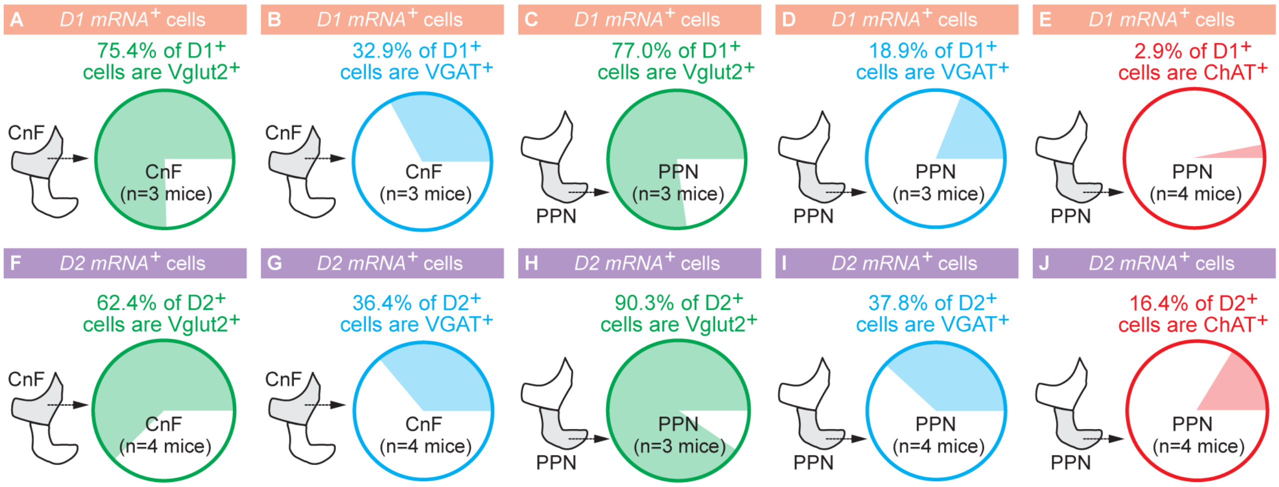

Figure S7. Proportions of *Vglut2 mRNA*$^+$, *VGAT mRNA*$^+$ and *ChAT mRNA*$^+$ neurons among D_1 or D_2 receptor-expressing neurons in the cuneiform nucleus (CnF) or pedunculopontine nucleus (PPN). Related to Figure 7. (A-B) Proportions of *D_1 receptor mRNA*$^+$ cells in the CnF expressing *Vglut2 mRNA* (A, 18/21 *D_1 receptor mRNA*$^+$ cells in mouse 1, 58/70 in mouse 2, 36/63 in mouse 3) or *VGAT mRNA* (B, 13/29 *D_1 receptor mRNA*$^+$ cells in mouse 1, 14/51 in mouse 2, 10/31 in mouse 3). **(C-E)** Proportions of *D_1 receptor mRNA*$^+$ cells in the PPN expressing *Vglut2 mRNA* (C, 31/48 *D_1 receptor mRNA*$^+$ cells in mouse 1, 54/64 in mouse 2, 58/70 in mouse 3) or *VGAT mRNA* (D, 3/33 *D_1 receptor mRNA*$^+$ cells in mouse 1, 12/49 in mouse 2, 9/38 in mouse 3) or *ChAT mRNA* (E, 0/17 *D_1 receptor mRNA*$^+$ cells in mouse 1, 1/22 in mouse 2, 1/34 in mouse 3, 2/45 in mouse 4). **(F-G)** Proportions of *D_2 receptor mRNA*$^+$ cells in the CnF expressing *Vglut2 mRNA* (F, 20/48 *D_2 receptor mRNA*$^+$ cells in mouse 1, 47/65 in mouse 2, 23/38 in mouse 3, 15/20 in mouse 4) or *VGAT mRNA* (G, 15/51 *D_2 receptor mRNA*$^+$ cells in mouse 1, 35/113 in mouse 2, 23/56 in mouse 3, 33/81 in mouse 4). **(H-J)** Proportions of *D_2 receptor mRNA*$^+$ cells in the PPN expressing *Vglut2 mRNA* (H, 58/61 *D_2 receptor mRNA*$^+$ cells in mouse 1, 25/30 in mouse 2, 12/13 in mouse 3) or *VGAT mRNA* (I, 21/35 *D_2 receptor mRNA*$^+$ cells in mouse 1, 28/83 in mouse 2, 15/41 in mouse 3, 18/84 in mouse 4) or ChAT mRNA (J, 3/36 *D_2 receptor mRNA*$^+$ cells in mouse 1, 8/61 in mouse 2, 17/87 in mouse 3, 18/79 in mouse 4). In A-J, quantifications were made from 1 to 3 slices per mouse.

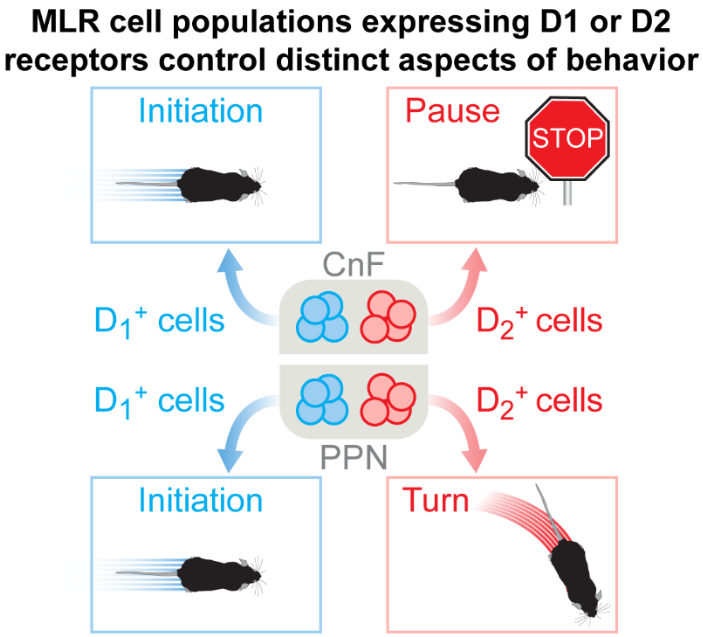

Figure S8. Behavioral roles of the dopamine-sensitive neurons in the mammalian Mesencephalic Locomotor Region (MLR). Related to Figure 2.
